# A Review on Heat Treatment of Cast Iron: Phase Evolution and Mechanical Characterization

**DOI:** 10.3390/ma15207109

**Published:** 2022-10-13

**Authors:** Ojo Jeremiah Akinribide, Olasupo Daniel Ogundare, Olanike Mary Oluwafemi, Kelechi Ebisike, Abdulganiyu Kehinde Nageri, Samuel Olukayode Akinwamide, Fehmi Gamaoun, Peter Apata Olubambi

**Affiliations:** 1 Center for Nanomechanics and Tribocorrosion, School of Mining, Metallurgy and Chemical Engineering, University of Johannesburg, Johannesburg 2000, South Africa; 2 Engineering Materials Development Institute, Ondo Road, Akure 340110, Nigeria; 3 Department of Mechanical Engineering, Aalto University, 02150 Espoo, Finland; 4 Mechanical Engineering Department, College of Engineering, King Khalid University, Abha 61421, Saudi Arabia

**Keywords:** morphology, phase analysis, mechanical properties, cast irons, austempered ductile iron, gray cast iron and ductile irons

## Abstract

The isothermal heat treatment process has been identified as a unique process of fabricating exceptional graphite cast iron due to its remarkable mechanical properties, such as excellent machinability, toughness, and high level of ultimate tensile strength. Austempered ductile iron (ADI), ductile iron (DI), and gray cast iron (GCI), known as spheroidal cast irons, are viable alternative materials compared to traditional steel casting, as well as aluminum casting. The graphite nodules from the microstructures of DI, ADI, and GCI are consistently encompassed by acicular ferrite and carbon-saturated austenite in the matrix, forming a distinctive ausferritic structure. All these materials are extensively used in the fabrication of engine sleeves, engine blocks, valves, gears, and camshafts in the automobile sector. With relative motion and outward loads, these components are regularly exposed to surface contact. In this project, it was observed that austempering temperature and a shorter holding period could also be used to manufacture needle-like ferrite platelets for austempered ductile iron (ADI) and other graphite cast irons. To overcome the brittleness challenges and catastrophic failures encountered by applied loads in present-day applications, it is essential to comprehend the isothermal treatments, morphological behaviors, phase analyses, processing techniques, and mechanical properties needed to properly incorporate these materials into future designs. This review article provides detailed information on the characterization and relevant potential mechanisms of ADI, DI, and GCI.

## 1. Introduction

**Figure 1:**
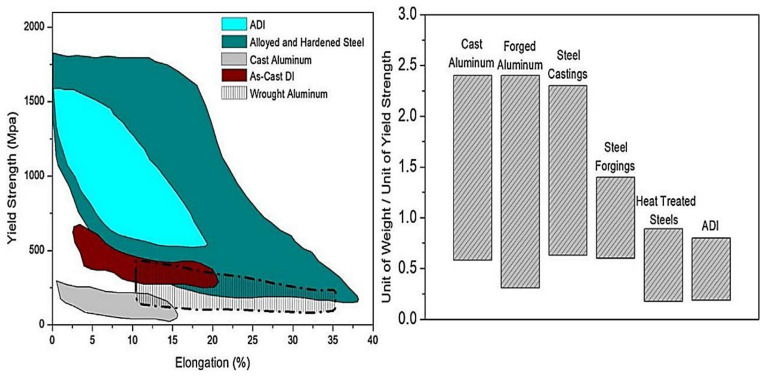
Graphite cast irons compared to similar engineering materials in terms of yield strength, elongation, unit weight, and unit cost [13].

Research has proved that gray cast irons, ductile irons, and austempered ductile irons are ferrous cast materials that have undergone different austempering heat treatment conditions, resulting in the production of new materials that have high strength-to-weight ratios with very tough and strong properties. Numerous casting techniques, such as green sand, bonded sand, lost foam, lost wax, continuous casting, centrifugal casting, and even permanent mold, can be used to create all these classes of cast irons. One of the ways of fabricating gray cast iron materials is using an isothermal process of heat treatment in a foundry shop. The properties obtained from these cast iron materials have proved their potency in mechanical properties when compared with steel casting, steel forging, aluminum, and even titanium made through traditional processing routes [1,2,3,4]. The nodular graphite is consistently surrounded by acicular ferrite and austenite saturated with carbon in austempered ductile iron, forming a distinctive ausferritic structure. Graphite cast irons are extensively used in the fabrication of many automobile parts, such as engine crankshafts, engine gears, and engine valves. With relative motion and external load, these components are often exposed to surface contact. The yield, or ultimate tensile strength, of any cast iron product, as seen in Figure 1, has been shown to be three times greater in value than alternative materials, such as cast and wrought aluminum, with fewer ductile properties. Despite the fact that hardened steel has higher maximum yield strength and elongation than ADI, the weight per unit of yield strength of ADI is lower than that of other aluminum and steels due to the presence of graphite nodules in the matrix. According to Mandal et al. [5], the cost of producing cast iron materials, such as austempered ductile iron, gray cast iron, and ductile iron, in terms of unit length was 20% more than the cost of producing steel castings and roughly the same as steel forgings and heat-treated steels. Furthermore, the cost of producing titanium alloys was higher than DI, ADI, and GCI, despite having a greater range of elongation under acceptable yield strength than graphite cast irons. In addition, titanium alloys have a high cost of production and poor moldability, making them unsuitable for industrial use. Aside from these, ADI has high levels of toughness, resistance, and machinability properties [6,7].

The unusual structural integrity of ausferrite, or carbon saturation in the austenitic phase, and the acicular ferritic characteristics of the material might all play a role in the outstanding mechanical properties established. In the last fifteen years, researchers have focused on phase-change and mechanical features of graphite cast irons utilizing various chemical contents and heat treatment methods, as well as developing mathematical algorithms [8,9,10,11,12].

**Figure 2:**
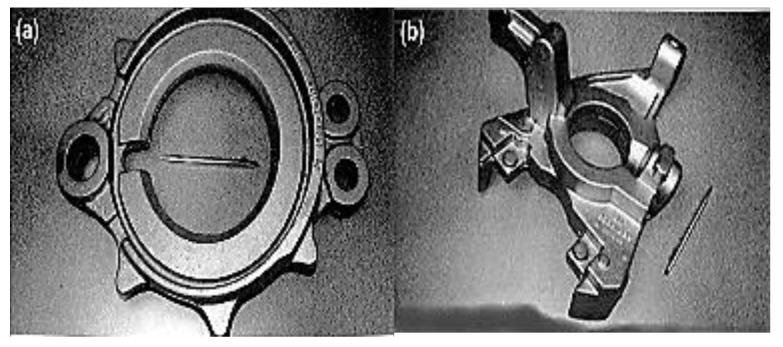
Graphite cast iron applications: (**a**) truck steering knuckle and (**b**) truck brake spider [14].

Graphitized cast iron materials have consistently served as a substitute for some conventional steel and aluminum in the manufacturing of commercial truck and car components, such as camshafts, crankshafts, gears, and engine valves, since the early 1970s [14,15,16,17,18], as presented in Figure 2. The bulk of these parts come into contact with the surface due to relative motion and external force. As a result, graphite cast iron’s high wear resistance is essential for trouble-free operation and a long service life.

Different scientists have examined the phase transformation and mechanical characteristics of graphite cast iron materials, and articles have been published that outline their performances in engineering service environments [13,19,20]. Nevertheless, there are still gaps in the information regarding the morphological structures, processing techniques, phase analysis, and overall mechanical behavior, which are discussed in this paper.

### 1.1. Production of Ductile Iron

**Figure 3:**
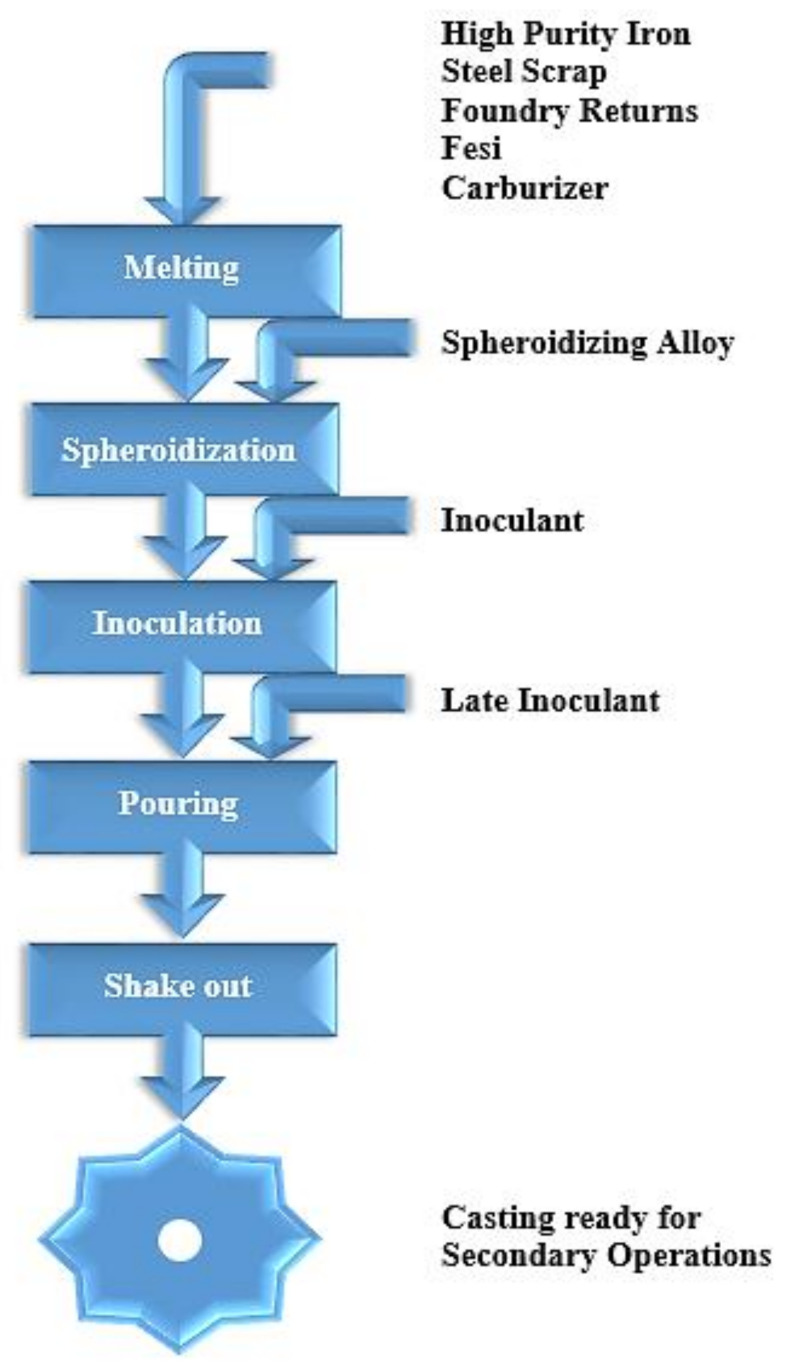
Schematic flowchart of ductile iron production [21].

Figure 3 shows a flowchart of ductile iron production. According to Trudel and Gagne [21], the charge materials must be cautiously chosen to avoid infiltration of the castings by specific alloying elements inherent in steel and to sustain a high standard of microstructure uniformity. The suggested limits for some of the most frequent harmful residual elements detected in DI castings are shown in Table 1 (e.g., S, P, Mn, Cr, and Cu). Although all of the production steps listed in Figure 3 are critical for producing flawless castings, special consideration is needed for the spheroidization and inoculation techniques, which regulate the crystallization of spherulitic graphite particles. To acquire high-quality DI components, the highest feasible levels of these must be obtained [21].

### 1.2. Composition of Ductile Iron

In gray iron castings, the weakest structural characteristics are graphite flakes, but in DI castings, defects are formed by elements seen in the charge materials (e.g., eutectic and interstitial carbides, steadite, flake graphite, etc.) that affect its efficiency. Table 1 shows the required chemical composition ranges for ferritic and pearlitic DI grades. Hardenability-boosting elements, such as Cu, Ni, Mo, and Mn, need to be alloyed in ductile iron castings for ADI manufacturing. However, Mo is often chosen over Mn due to its greater hardenability improvement impact and analogous intercellular carbide formation propensity.

### 1.3. Microstructures and Properties of Ductile Iron and Austempered Ductile Iron

**Figure 4:**
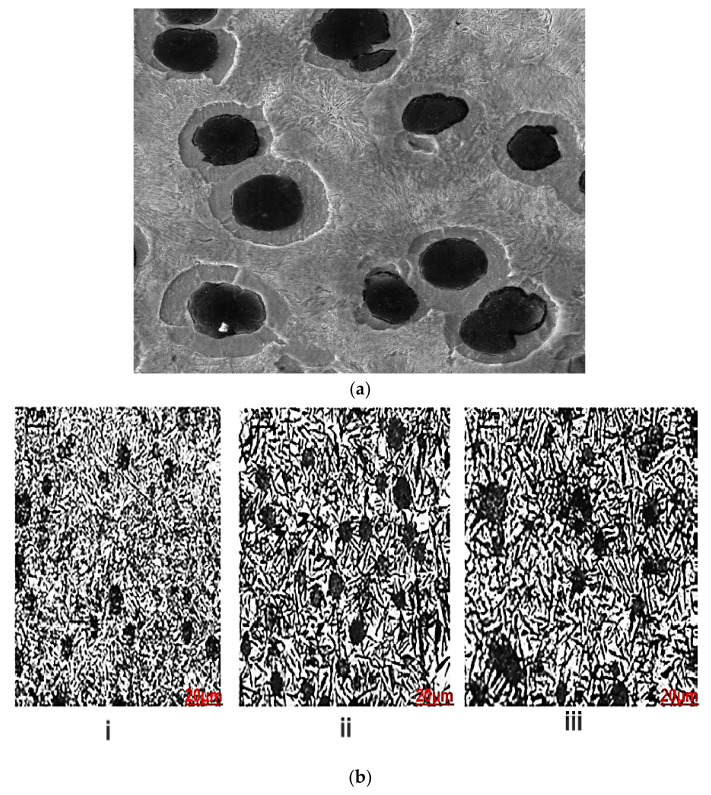

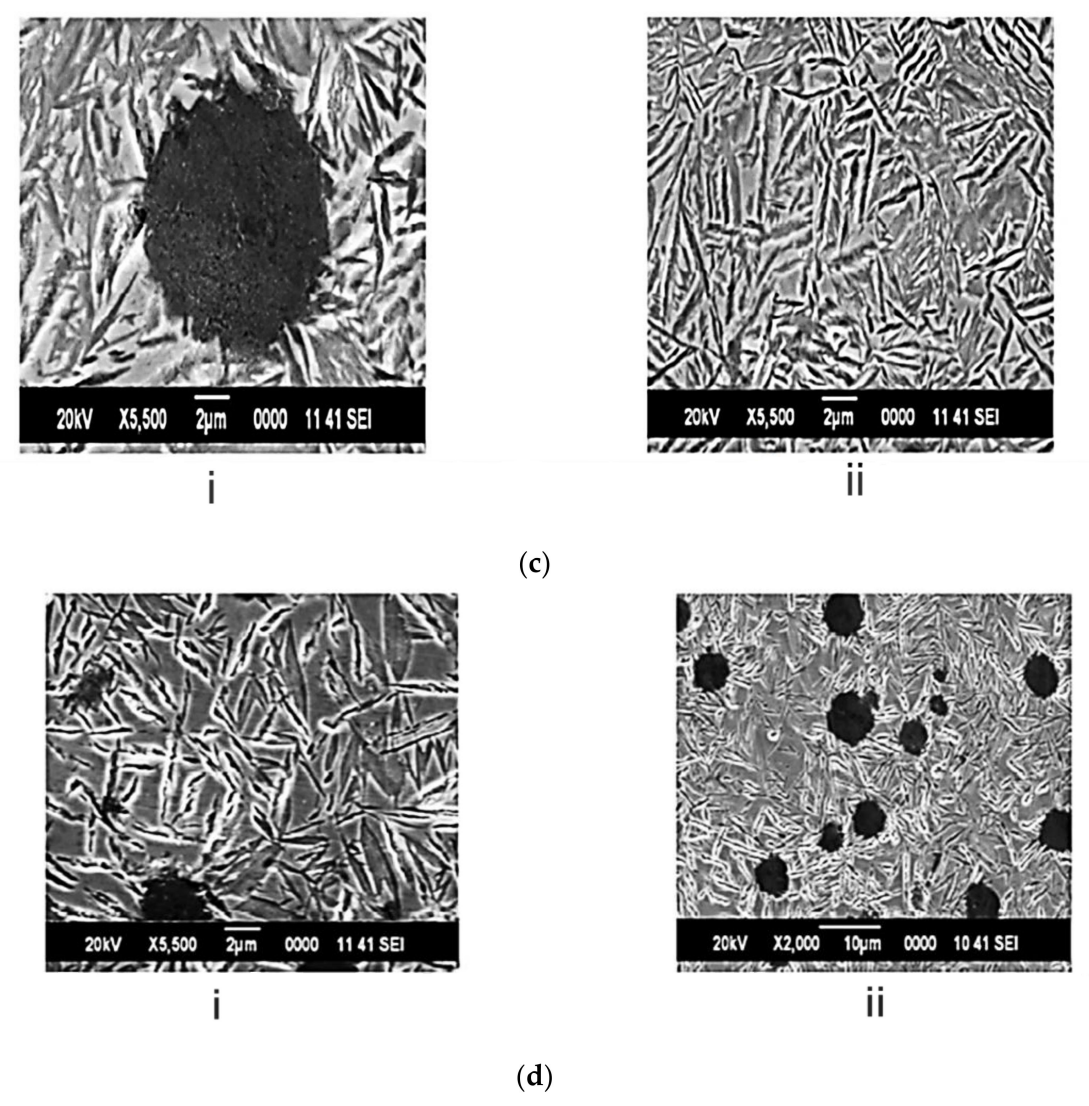
(**a**) Unetched microstructure of ductile iron. (**b**) Microstructure of 2 mm thick plate austempered at 400 °C; kept for (**i**) 2 min, (**ii**) 5 min, and (**iii**) 10 min; and inoculated with Ce-Ca-Al-S-O-FeSi at 0.4%. (**c**) SEM micrograph of 2 mm thick plate austempered at 400 °C and kept for 2 min (**i**) around nodule and (**ii**) in matrix inoculated with Ce-Ca-Al-S-O-FeSi at 0.4%. (**d**) SEM micrograph of 2 mm thick plate austempered at 400 °C and kept for 10 min (**i**) around nodule and (**ii**) in matrix inoculated with Ce-Ca-Al-S-O-FeSi at 0.4%.

Ductile iron materials are distinguished by the presence of spheroidal graphite in the ferrous matrix, as seen in Figure 4a. The higher the number of spheroids (or nodule count), the better the casting quality: a high nodule count, which is essentially regulated by the inoculation process, inhibits chemical segregation during solidification and assures the structural uniformity of a component. Graphite nodules are mostly spherical, since uneven particles of graphite are observed at maximum stress, where a minimum of 80% nodularity is easily attained [21].

It is widely known that castings should be made with uniform graphite qualities, followed by modification of the mechanical properties by the matrix microstructure. The ferrite/pearlite ratio regulates the properties of a defect-free unalloyed casting in its as-cast state. However, the matrix structure can be modified by alloying or heat treatment to provide the required properties. Martensite is a solid solution of carbon in iron that is interstitially metastable. It is created when austenite is rapidly cooled to room temperature. At low carbon concentrations, it can have a bcc structure, whereas, at high carbon concentrations, it can have a body-centered tetragonal structure. Depending on the composition of the alloy, pearlite formation can start at temperatures between 1150 °C and 723 °C during the gradual cooling of iron alloys. Typically, ferrite and cementite are combined in a lamellar (alternative plate) structure (Fe_3_C). Despite possessing the same body-centered cubic crystal structure as ferrite, martensite is less stable than ferrite, as evidenced by the fact that the equilibrium austenite fraction in the recrystallized ferrite matrix is significantly smaller than that in the tempered martensite matrix.

The change of austenite to ferrite and graphite begins in the presence of graphite spheroids that serve as sinks for the rejected carbon atoms as the austenite cools below ferrite phase A1 in the iron–carbon phase diagram according to [22]. The ferrite region transforms into a sphere-shaped shell that envelops the graphite spheroid as the reaction progresses, producing the recognizable bull’s-eye structure. An incubation period is often attributed to the nucleation and development of ferrite to a detectable level, typically with 0.1% to 0.2% carbon atoms by weight, according to experimental studies of the isothermal kinetics of this reaction [22,23]. A rejection of carbon atoms that diffuse through ferrite shells to the existing graphite spheroids and austenite is accompanied by growth in ferrite shells.

Components made of austempered ductile iron (ADI) have excellent toughness (50–60 joules), high strength (1000–1500 MPa), decent ductility (4–12%), and great wear resistance. ADI parts are less expensive than identical forged steel parts and may result in weight savings of up to 10%. A two-phase mixture of austenite and acicular banitic ferrite makes up the novel matrix structure of austempered ductile iron (ADI).

It can be seen from the microstructures in Figure 4b(i)–(iii) that ausferrite and feathers of bainite with varying volume fractions make up the majority of the microstructures. The volume fractions of bainite and ferrite are slightly different, according to the observed microstructures. SEM images of a 2 mm thick plate that was austempered at 400 °C and held for 2 and 10 min were acquired for further analysis and are displayed in Figure 4c,d, respectively. These SEM images in Figure 4c show that the bainitic feathers are equally distributed in the matrix and surrounding nodules. The bainitic feathers appear to be the same size and form in Figure 4c,d, with a minor volume percentage of residual austenite surrounding them.

### 1.4. Wear Mechanisms of ADI

During wear tests, the graphite nodules could be damaged and spread off the wear track. The small graphite particles act as a dry lubricant, lowering the coefficient of friction and, thus, reducing wear loss. In the absence of a fluid lubricant, oxidative wear has been mostly reported as one of the main wear mechanisms. Due to high brittleness, the oxidative layer is easily broken during sliding motion. Furthermore, some oxidative wear debris (Fe_2_O_3_ and FeO) with high hardness may scratch or even tear up the base material [24,25].

#### Types of Wears

- Abrasive wear is a kind of wear mechanism that causes the material on the surface to disintegrate owing to the influence of a hard particle in contact with the surface. It can also happen when a hard surface or particles, such as DI, ADI, and GCI, interact with or slide on a soft surface, causing material loss [26]. This is a type of wear caused by the loading of a solid particle on the surface of a material with a hardness equal to or less than that of the loaded particle.
- Adhesive wear is when two bodies attach to one another locally, allowing material to be transferred from one body to the other. An example is sliding wear, which occurs when one solid moves across another. Galling wear is a severe type of adhesive wear. Scoring, or scuffing, wear occurs when grooves and scratches form in the sliding direction.
- Erosive wear is majorly caused by the impact of a stream of solid particles and is mostly determined by the particles’ size, hardness, velocity, and angle of impact. The wear rate normally increases as particle size, angularity, hardness, and impact velocity increase.

## 2. Graphite Cast Irons under Isothermal Heat Treatment

Graphite cast iron materials, such as gray cast irons, austempered ductile irons, and ductile irons, are classes of structural material that are being produced at an ever-increasing rate. They are generally considered among prospective construction materials because of their promising tensile properties, yield strength, thermal stability properties, and affordable cost. These graphite cast irons are utilized in a variety of industries, including machine manufacturing and nonparasitic load-bearing equipment in the defense sector. They are primarily used in the casting of high-precision load-bearing components, such as crankshafts for heavy-duty vehicles, oil industry pressure pipes, and so on [27,28]. The specific matrix structure, dominated by acicular ferrite and austenite, provides graphite cast irons their distinguishing feature, known as ausferrite [27,28]. This matrix is frequently referred to as bainite, according to reports from researchers, even though carbides are not present in the structure [25]. Their structures are specially developed by the meticulously regulated thermal treatment of nodular cast iron [29,30].

**Figure 5:**
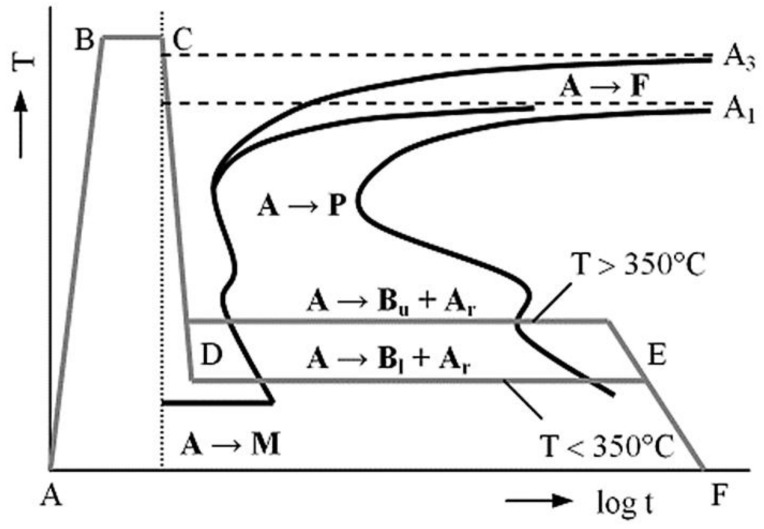
Isothermal heat treatment of the designated heat treatment sections (A—austenite, F—ferrite, P—pearlite, B_u_—upper bainite, B_l_—lower bainite, A_r_—retained austenite, M—martensite) [30].

The stages for austempering include: heating to the austenitization temperature (AB); holding time at the austenitization temperature (BC); quick cooling to the temperature of isothermal transformation of austenite (CD); holding time at this temperature (DE) until austenite is changed into bainite; and dropping the temperature to ambient conditions (EF), which is normally performed gradually to avoid stress accumulation, as seen in Figure 5 [31].

## 3. A Review on Processing Techniques of Austempered Ductile Irons

**Figure 6:**
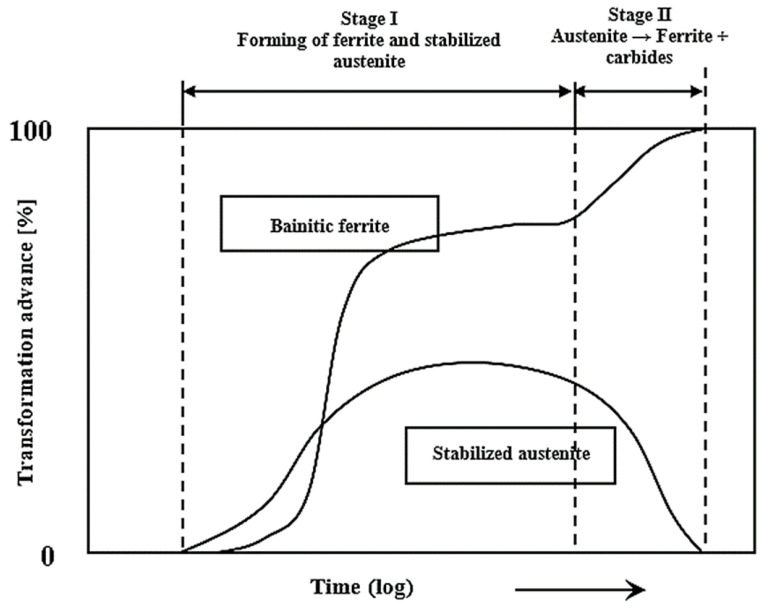
Austempered gray cast iron’s phase transformation as a function of austempering time and temperature (T = 300–350 °C) [32].

Kaczorowski et al. (2007) [32] in their research on the mechanical properties and structure of ADI indicated a report as follows: EN-GJS-500-7-grade ductile iron was chosen for the investigation, while the heating process was conducted in a three-ton induction furnace. Prolonged heating at a temperature below the eutectoid temperature was performed with an FeSiMg alloy in a tightly covered ductile iron treatment ladle in order to improve the mechanical properties. The FeSi75 alloy was introduced to the melt before pouring into the prepared sand molds. After the castings, a test specimen for a tensile test was cut to a 4 mm diameter for further treatment. The samples were later subjected to a heat treatment process for 1 h at 900 °C to cause one or more constituents to enter into solid solution and then rapidly quenched for case-hardening purpose in Figure 6. This was followed by a further heat treatment process called austempering, which was carried out at varying temperatures of 275 °C, 300 °C, 325 °C, and 350 °C. The varying times also included 15, 45, and 90 min for all the austempering temperatures. Meanwhile, 30 and 180 min were also considered for the temperatures of 275 °C and 300 °C.

The test samples were prepared for further testing after the austempering treatment. Proper grinding of the surface layer was carried out up to a thickness of 0.2 mm in order to remove the carbon depletion surface caused by the prior heat treatment process. A tensile test was conducted with Instron 1115, and the mechanical properties were assessed and evaluated for the three test samples representing each heat treatment parameter used. Surface hardness was measured in Brinell hardness number (BHN), and a 2.5 mm steel ball loaded at 15 s with a force of F = 187.5 N was applied accordingly. Additionally, light microscopy and surface morphology was carried out on the prepared test piece using SEM and TEM for proper investigation. The samples for microstructural analysis were prepared by grinding and polishing them with an appropriate grinding machine before etching with a 4% HNO_3_ solution in COH. The structure was viewed under an Olympus IX-70 light microscope (Shinjuku, Japan) with different interpretations for assigned magnifications. A Hitachi scanning electron microscope (Tokyo, Japan) was used, and the pU = 20 kV. The preface slices were properly prepared for capturing using the thin foil method. To prepare the sample, rods of 3 mm diameter were cut from the bulk, 0.1 mm thick disks were later cut with a wire saw (IF-07A) of lesser load, and a Gatan precision ion-polishing system was later employed. A Philips EM 300 microscope (Amsterdam, The Netherlands) operating at 100 kV was used to view the thin foil. Meanwhile, the measurement of the sample tilt was in the range of ±45°, which was performed using a goniometer. From the report, it was discovered that an increase in austempering time caused a decrease in ductility, while the ductility increased with decrease in the austempering time. In addition, as the austempering temperature decreased, the hardness of the ADI test castings increased. This result showed an improvement in the mechanical properties of the materials. Considering the lowest austempering temperature at 275 °C, the results showed an apparent increase in the hardness. Additionally, the highest austempering time (180 min) resulted in an obvious increase in the ductility of the test piece due to plastic deformation. The strengthening process increased the numbers of dislocations, thereby increasing the hardness and exhibiting more ductility, as seen in the face-centered cubic crystal structure. Table 2 show grade identification of ADI and their mechanical properties

Alabi and Aluko (2013) [33] reported ductile cast iron produced from bits and pieces of engine block obtained from the remains of gray cast iron. An amount of 55 kg of this was loaded into a furnace with 4 kg of graphite, 700 g of ferrosilicon, and 47 g to 326 g ferrosilicon manganese. It was inoculated with 18 g of powdered graphite and ferro-silicon, respectively. A mixture of engine oil and diesel with the help of a blower was used to fire the furnace, and a rotary furnace located at the Engineering Materials Development Institute, (EMDI) Akure, Nigeria was used to melt all the charge appropriately. After casting, the ductile samples were austenitized in a salt bath mixture of potassium (K), sodium (Na), and barium (Ba) chloride in a ratio of 3:2:1, respectively, at 830 °C for 120 min, in addition to being held for extra minutes. Thereafter, the specimens were rapidly quenched in a molten salt bath composed of sodium and potassium nitrate salts (NaNO_3_ + KNO_3_) in a 50:50 ratio. The austempering temperature was maintained at 450 °C before cooling to room temperature to allow the required ferrite and austenite structure to form. Thermocoupling (AVD890C) was used as temperature sensor to monitor the salt bath temperature all throughout the process. The ductile iron and ADI produced were examined critically to learn the chemical composition appropriately. The results obtained from the casting and austempering processes revealed that the addition of ferromanganese significantly reduced the amount of sulphur present in the melt; this was a result of the combination of sulphur and ferromanganese, which produced manganese sulphide. Meanwhile, because of the low density of the manganese sulphide, it was dispersed in the face of the melt and later tapped as slag. The results also confirmed the process of producing ductile iron with an optimum sulfur level and the modification of ductile iron to austempered ductile iron. It also established a suitable time for the inoculants to be introduced into the melt, which was when the metal was about to be poured into the ladle. It was seen that all the materials charged into the furnace, as well as all the material, completely turned to molten metal within 40 min. It was established that the results conformed to the universal standard in the literature.

Prabhukumar Sellamuthu et al. (2018) [34] also reported their research, where the materials were calculated appropriately and charged directly into an electric furnace (induction). After melting, the molten metal was poured into a mold prepared according to the dimensions needed for further testing. Following casting in the shape of a Y-block, a sample with a 20 mm thickness in dimension was sectioned out for further testing. Thereafter, the specimens were subjected to spark analysis to learn the chemical composition of the casting. This analysis was performed using an optical emission spectrometer (spark). The samples were first preheated for 10 min in a pre-heating furnace at 450 °C to remove grease and debris from the surfaces of the samples. The samples were then heated to 840 °C for 30 min in a conserved austempering furnace before rapidly quenching in a salt bath composed of sodium (Na) and potassium (K) nitrate salts. This was followed by a further heat treatment process called austempering, which was carried out at varying temperatures of 300 °C, 320 °C, 340 °C, and 360 °C to examine the effect of austempering temperature on mechanical properties and microstructural development. Lower austempering temperatures were chosen in order to prevent the development of bainitic or martensitic structures.

**Figure 7:**
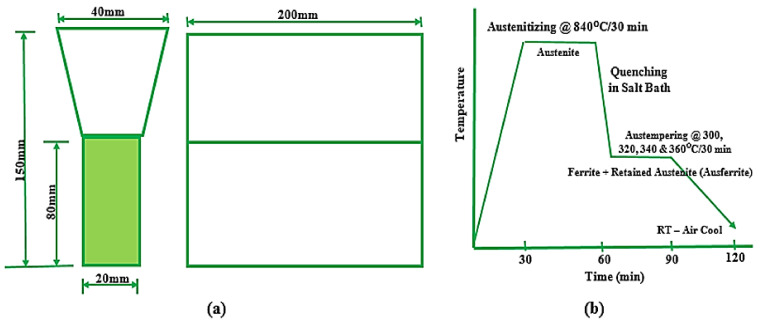
(**a**) Y-block shape and (**b**) stages involved in austempering of ADI [34].

Before subjecting the samples to further mechanical testing, they were thoroughly washed in hot water using washing machine for about 30 min to eliminate the salt on the face of the specimens. A tension test, an impact test (Charpy), and a hardness test were carried out on the heat-treated and control samples in order to investigate the influence of the austempering process in Figure 7. The samples were also prepared for microstructural analysis. The selected portions were cut and ground with different grits of emery paper and polished to a shining, mirror-like surface before etching. The etchant used was 2% nital, and the microstructures were viewed under an optical microscope. Micrographs of the unetched samples were captured at 100× magnification, while those of the etched samples were captured at 400× magnification. The results were then compared and evaluated based on the phases present in the microstructures. From the results, the micrographs showed a more coarsened structure and higher quantities of retained austenite (softer phase) as the austempering temperature rose from 300 °C to 360 °C; this drastically reduced the hardness and strength values. A finer microstructure was obtained at the lower austempering temperature (320 °C), which resulted in higher tensile strength. As the quantity of the retained austenite rose, the impact energy also rose.

Zammit A 2019 [35] reported in his research work as follows: Test specimens (5 mm diameter pins) were produced from a casting made of ductile iron. In order to avoid loss of carbon on the surfaces of the materials during the heat treatment process, the samples were painted with SEMCO paint and later heat-treated to 900 ± 2 °C (austenitized) for 60 min. Quenching was performed promptly in a salt bath at 360 ± 5 °C, and the material was allowed to cool in air to an ambient temperature. In addition, test pieces for the disks were prepared from tool steel (90 mm diameter each). Aiming at improving on the hardness, these samples were first preheated before being heat-treated to 1025 °C and rapidly quenched with nitrogen, while the pressure was 5 bars. The tempering process was carried out at 190 °C for 180 min. Subsequently, the pins and disks were perfectly prepared for the shot-peening process after the heat treatment processes. An S330 shot-peening machine was used, and all the necessary precautions at each stage were followed to detail for a good outcome. Thereafter, a Mitutoyo (MVK-H12) microhardness tester was used to determine the hardness, and an X-ray diffraction technique was used to determine the residual strain and to evaluate the phases appropriately. It was discovered from the results that the microstructure of the austempered materials revealed an intergranular arrangement of fine, interlocking grains. This was also characterized by high angle boundaries between ferrite grains, and also, there was substantial austenite retention. This structure was recognized to improve on steel properties, especially the toughness. There were improvements in the microhardness of the ADI structure (336 ± 15 HV) and that of the ausferritic structure (370 ± 10 HV). Shot peening of the austempered ductile iron resulted in transformation-induced plasticity (TRIP). High plastic deformation during cold working changed the austenite structure to a martensitic structure as a result of shot peening on the surface of the material. There was an increase in the hardness, and the high hardness was due to work hardening and stress-induced austenite changing to martensite. Meanwhile, the hardness at the surface was higher the one inside the material.

Wilson Sckudlarek et al. 2021 [36] also reported in their research that an induction melting furnace (380 kg) was used for the melting of ductile iron (grade 800-55-06) weighing 350 kg at 1520 ± 10 °C. This was nodularized with FeSiMg (8% Mg), and the inoculant used was FeSi75 (75% Si). A sample in the form of a coin was obtained in order to evaluate the chemical composition. A Bakelite mold was prepared in the form of a Y-shape, and half of the melt was poured at 1355 ± 15 °C, while niobium (Fe-65Nb) with particle sizes ranging from 1 mm to 5 mm was introduced to the remaining part of the melt in the furnace as the temperature increased to 1520 ± 10 °C. To allow proper homogenization, it was left for 15 min before pouring. The cast was properly cleaned and made into tensile test pieces of 65 mm lengths and 8 mm diameters, according to ASTM E8/E8M-13. Samples were also prepared using the ASTM E3-11 standard for microstructural analysis. All the prepared samples were first pre-heated at 450 °C for more than 4 h before being heat-treated at 900 °C, soaked for 2 h, and rapidly quenched in a salt bath at varying temperatures of 320 °C and 360 °C and varying times of 15 min, 30 min, 1 h, and 1 h 30 min. The tension test was carried out at ambient temperature with a universal testing machine (INSTRON, EMIC 23-300-DL30000, Norwood, MA, USA). The capability was 300 kN, and the rate of testing was 10 mm/min. Subsequently, an optical microscope and scanning electron microscope were used to view the microstructure before and after etching, and the etchant used was a 4% nital solution. Additionally, an object or image analyzer was used, and the graphite nodules were evaluated using the ASTM A247-10 standard; with this, the number and size distribution of the graphite nodules, the degree of nodularization, and the shape of the nodules were analyzed. The amount of niobium in each phase was evaluated accordingly. It was discovered from the results that the addition of niobium to ductile iron influenced the mechanical properties positively. The presence of 0.35%Nb and the austempering process increased the ultimate tensile strength and yield strength, but this resulted in a reduction in the percentage of elongation. Refinement of the graphite nodules modified the microstructure such that the number of nucleation sites available for the growth of graphite nodules was significantly improved. It also showed that all the austempered ductile iron samples and the alloyed one (0.35%Nb) gave higher hardness and strength values at the lower austempering temperature (320 °C) but lower ductility due to plastic deformation during the strengthening process. The alloyed sample (ADI 0.35%Nb) austempered at both austempering temperatures (320 °C and 360 °C) greatly improved the machinability. Invariably, the heat treatment process on the ADI samples with the austempering temperature of 360 °C for 1 h presented the minimum results, which agreed with the ASTM A897-16 standard as stated by the Quality Index.

Table 3 shows the various types of austempered ductile iron that can be formed by heat treatment. It is shown from the Table 3 that an austempering treatment applied to ductile iron can increase its yield strength, elongation, and ultimate tensile strength.

**Figure 8:**
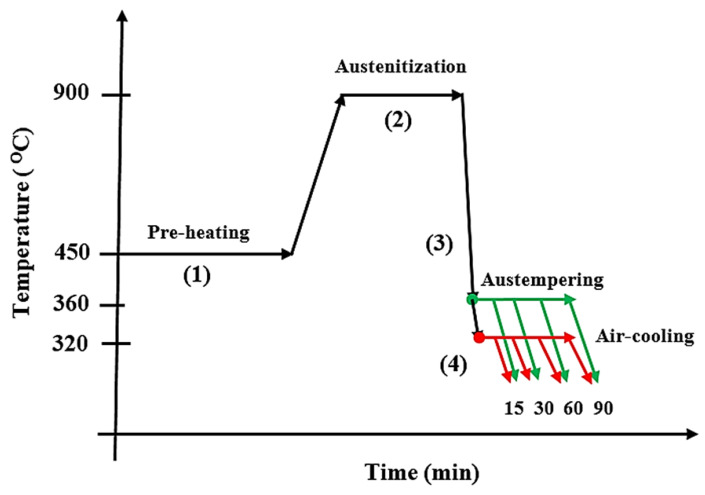
Graphical representation of the austempering heat treatment process of tested alloys [36].

Xue Han et al. 2020 [37] in their research reported that test specimens (arc disks) with dimensions of 7.6 mm in length and 63 mm in width were cut from ductile cast iron. The ductile iron samples were produced via a sand-casting process. All the disks (test specimens) were austenitized at 832 °C for 20 min in a molten salt bath furnace to allow the uniformity and homogeneity of the matrix. The samples were later austempered in a salt bath furnace for 2 h at varying temperatures of 232 °C, 288 °C, and 398 °C in Figure 8. Lower the austempering temperature and adequate time was observed in order to produce an ausferritic matrix and a carbon-enriched austenite, and the specimens were cooled in oil to an ambient temperature. Hardness testing was conducted on all the austempered samples to discover the influence of the strengthening process on hardness.

**Figure 9:**
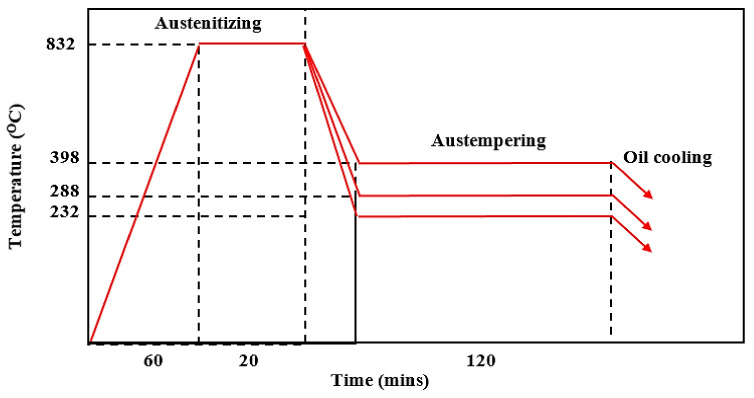
Austempering heat treatment process for ductile irons [37].

In addition, the samples were prepared for a laser surface-hardening process, and grinding and polishing were carried out on the samples using abrasive papers (grit size 240–1200). Surface features such as roughness and flatness were studied with a NanoMap 3D contact profiler. Subsequently, laser surface hardening was carried out on the samples as follows: The current used was 120 A, while the pulse speed was 2 mm/s; the laser spot was 2 mm with an 8 mm pulse width. Meanwhile, the outcomes of laser hardening on the wear of the ADI at varying temperatures of 232 °C, 288 °C, and 398 °C were examined at a 4 mm gap between laser-hardened patterns in Figure 9.

Tribological experiments were carried out on the specimens using a reciprocating Universal Macro Materials Tester (UMT-3) from Cetr, inc. 1715 dellave, campbell, Canada. The average coefficient of friction and wear were measured and recorded accordingly using the following parameters: A ball made of aluminum with a 4 mm diameter was used. The hardness of the ball was 75HRC, and the load used was 400 N, while the distance was 10 mm. The time used was 50 min with a test speed of 2 Hz, and the base oil was PAO4. The tests were repeated for three times on all the surface-hardened austempered ductile iron samples and the control samples, and the data obtained with the tribotester were used for further analysis. The samples were later cleansed with acetone, and the worn surfaces were viewed with a scanning electron microscope. From the results, identical wear marks and shapes were revealed for the entire test carried out for all the test conditions. Insignificant wear loss was discovered at the highest-temperature region saturated with iron carbide and cementite with the highest hardness value. As the phases transformed from martensite (hard) to tempered bainite (soft), the wear loss increased slowly. Additionally, the effect of the laser-hardened samples showed less wear lost because of the enhanced surface hardness as a result of the hardening process. An increase in the austempering temperature resulted in a progressive increase in the wear loss at the tempered bainitic zone; this was because of the lower hardness at this zone. Lesser wear volume was also discovered in the laser-treated samples than the untreated ones because of the laser-hardened layer. Thus, the treated samples had a more enhanced anti-wear performance than the control samples.

Zammith et al. 2019 [35] also reported in their research some test samples produced from Cu-Ni ductile iron that were heated to 900 ± 2 °C (austenitized temperature) and held for 120 min to allow uniform transformation to take place. The samples were rapidly quenched in a hot bath of molten salt at 360 ± 5 °C (above the Ms temperature of the alloy) and soaked for 60 min. The samples were later cooled in air to an ambient temperature. A carbon dioxide laser was used for the surface hardening in this research work; the wavelength was 10.6 µm with a focal length of 190.5 mm. A wear test was later conducted on the samples to measure the friction coefficient (COF) using a tribometer (pin-on-disk). The pins were cylinders of 5 mm in diameter made from austempered ductile iron, while the complementary disks were also machined from heat-treated steel with hardness values up to 60 HRC (AISI D2). The base oil was AGIP Rotra LSX 75W-90. The oil was first used to lubricate the disks, which were then rotated at 600 rpm for 5 s to wipe away the lubricant before the commencement of the test.

**Figure 10:**
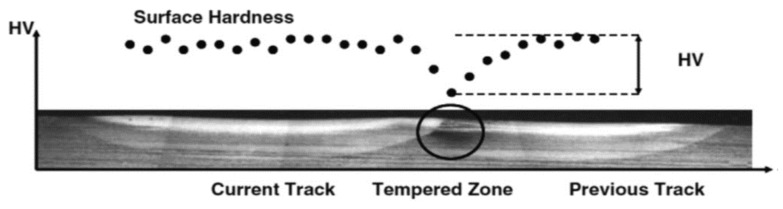
Back-tempering process in laser hardness [35].

Following the standard procedure, the tests were conducted at room temperature with a relative humidity of 42.4 ± 1.6% using the following test parameters in Figure 10: The pressure used was 10 Mpa, and the speed at which it rotated was 1450 rpm with 10 Mpa. The rotation was performed repeatedly up to five times per sample to the point at which scuffing occurred and was discovered. Subsequently, fatigue tests (rolling contact) were conducted on the samples, where a modified version of a T-03 four-ball wear tester was used. The test load was 300 N; the base oil was Shell transaxle gear oil 75W-90; and the rotational speed was 1450 rpm. The steel ball bearing (12.7 mm) was 100Cr6 chrome alloy, and the roughness value R was 0.032 µm. The test was performed at room temperature 22 ± 3 °C and at a relative humidity of 42 ± 4%. The data obtained from the test were analyzed using life data analysis contact. In addition, a scanning electron microscope (Carl Zeiss Merlin, Oberkochen, Germany) and a Nikon Optiphot-100 optical microscope (Minato ku, Japan) were used to view the surfaces of the specimens. The hardness values were examined in Vickers using a micro hardness tester (Mitutoyo MVK-H12, Kawasaki, Japan) with a test load of 50 g for 10 s. A profilometer (Mitutoyo Surf test 501) was also used to examine the surface roughness. From the results, the laser-hardened austempered ductile iron showed a better rolling contact fatigue (RCF) performance than as-austempered samples; this was a result of the optimum hardened surface hardness of ~770 HV, while that of the as-austempered specimen gave a lesser hardness value (~370 HV). It also showed that laser-hardening treatment enhanced the RCF lifespan of ADI. The laser hardening improved the scuffing and sliding wearing rates of ADI.

The resulting microstructure consisted of martensite with retained austenite on the laser-hardened samples, resulting in a better performance than the as-austempered samples. The results obtained affirmed laser surface hardening as an appropriate method for optimizing the mechanical properties of ADI.

## 4. Effect of Heat Treatment on Microstructure and Phase Evolution of ADI

According to David J.R., 2001, the significant effects of some major alloying elements on the austempering behaviour of austempered ductile iron are listed below [38]:

**Carbon:** Tensile strength was increased by adding carbon in the region of 3–4%, while elongation and hardness were barely affected. With the exception of instances where deviations were necessary to achieve a defect-free casting, carbon should be kept within the range of 3.6–3.8%.

**Silicon**: Silicon is one of the most crucial components in ADI because it encourages the synthesis of graphite, lowers the solubility of carbon in austenite, raises the temperature of the eutectoid, and prevents the synthesis of bainite carbide. The impact strength of ADI rose as silicon content rose, while the ductile–brittle transition temperature fell. Silicon should be tightly regulated between 2.4 and 2.8%.

**Manganese**: Manganese acted both as a helpful and a detrimental factor. While it segregated to cell boundaries during solidification where it created carbides and slowed the austempering reactions, it significantly boosted hardenability. As a result, Mn segregation at cell borders could be sufficiently high in castings with low nodule counts or section sizes bigger than 1/4 inch (19 mm) to result in shrinkage, carbides, and unstable austenite. The machinability and mechanical characteristics of these materials were decreased by these microstructural flaws and inhomogeneities.

**Copper**: To improve hardenability, ADI may receive up to 0.8%Cu. At austempering temperatures below 350 °C, copper increased ductility but had no discernible impact on the tensile characteristics.

**Nickel**: Up to 2% Ni may be used to increase the hardenability of ADI. For austempering temperatures below 350 °C, Ni reduced tensile strength slightly but increased ductility and fracture toughness.

**Molybdenum**: Molybdenum was the most potent hardenability agent in ADI and may be required in heavy-section castings to prevent the formation of pearlite [38].

**Figure 11:**
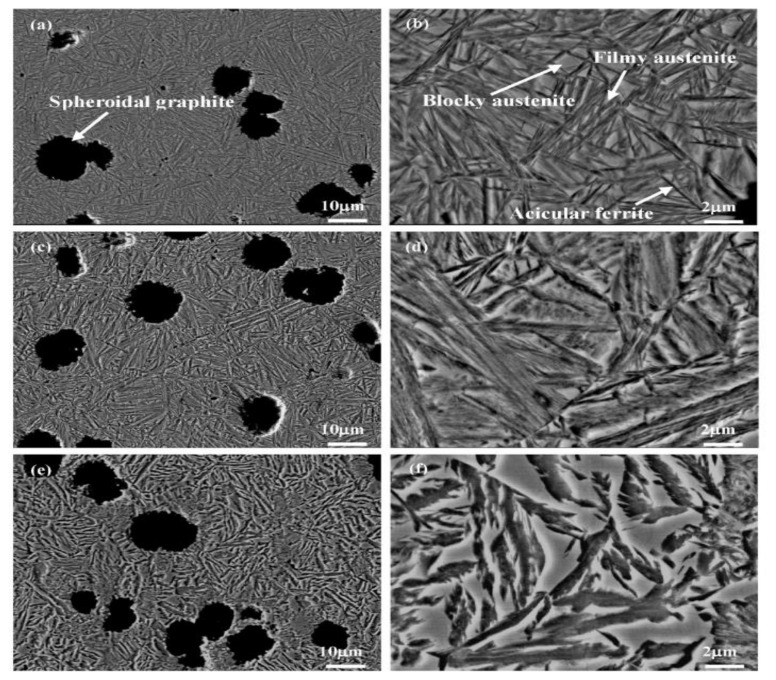

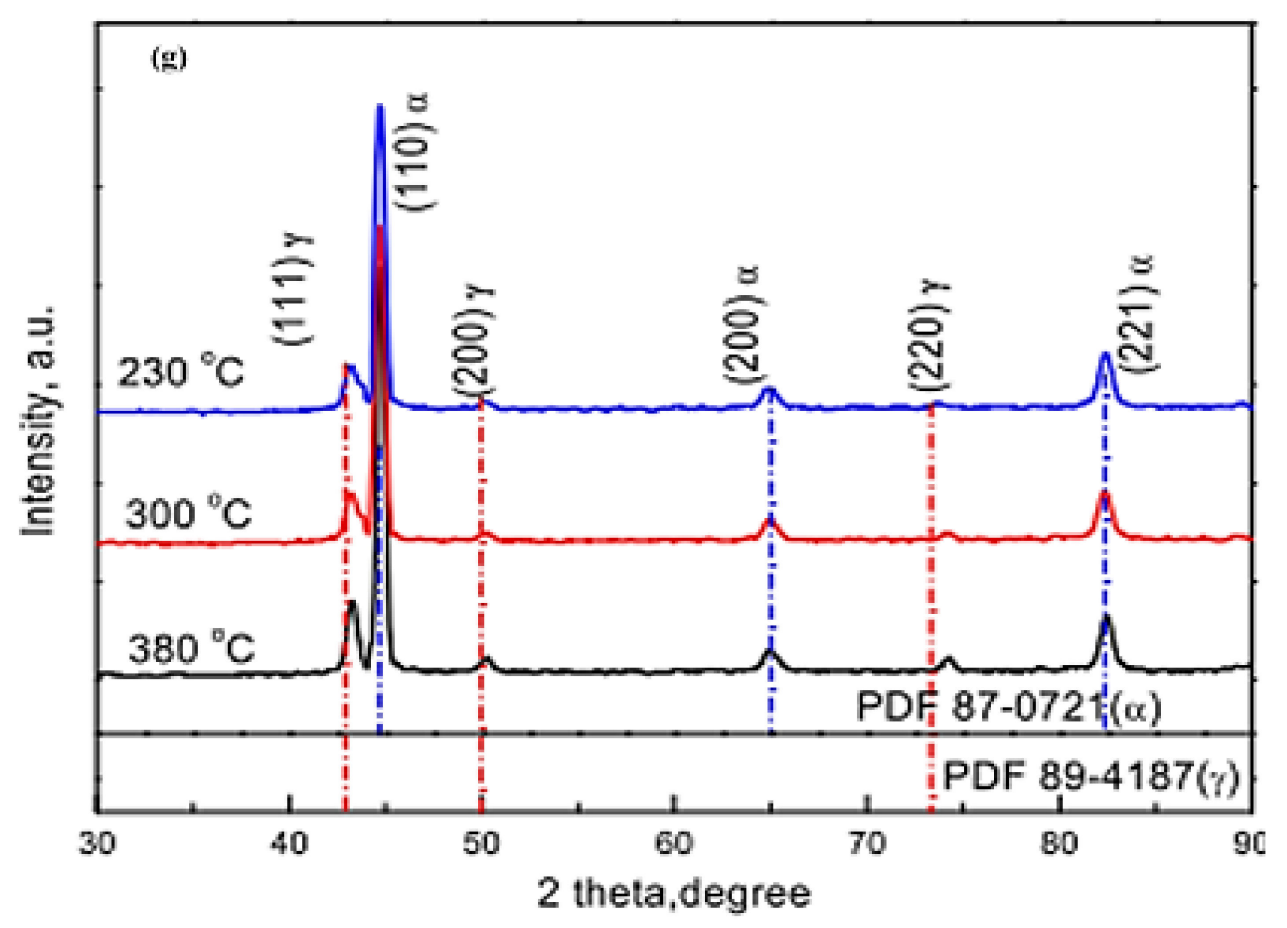
Secondary electron mode S.E of morphological images of the austempered specimens at (**a**,**b**) 230 °C, (**c**,**d**) 300 °C, and (**e**,**f**) 380 °C and (**g**) the phase analysis of the austempered ductile iron samples [13,39].

As reported by Du et al. (2020) [39], the nodularity in austempered conditions was identical for all ADIs, and no noticeable changes in the form and distribution of spheroidal graphite were found, as shown in Figure 11. Figure 11 shows the microstructural characteristics of graphite in ADIs at various temperatures. The graphite volume fractions and graphite counts in the ADIs were similar. Spheroidal graphite, acicular ferrite, and preserved austenite were all found in all three samples. Needle-like microstructures were obtained in the samples austempered at 230 °C (Figure 11b,d), whereas a feather microstructure was observed in the samples austempered at 380 °C (Figure 11f). The size of acicular ferrite grew as the austempering temperature increased (Figure 11b,d,f). Furthermore, two forms of preserved austenite were observed: one was blocky austenite, which was easily identified, and the other was filmy austenite distributed amongst acicular ferrite (Figure 11b). With increasing austempering temperature, blocky austenite grew while filmy austenite decreased. There was almost no filmy austenite identified in the sample austempered at 380 °C (Figure 11f). Moreover, the maximum points of austenite (γ) and ferrite (α) were revealed and showed that the matrix of ADI was made up of α and γ phases. ADI XRD patterns are shown in Figure 11g, although the relative strength of the α and γ phases varied, revealing that the number of α and γ phases was influenced by the austempering temperature. Observation revealed that the diffraction angle of the γ phases differed from the usual result, which was caused by the presence of supersaturated carbon in the γ phase.

**Figure 12:**
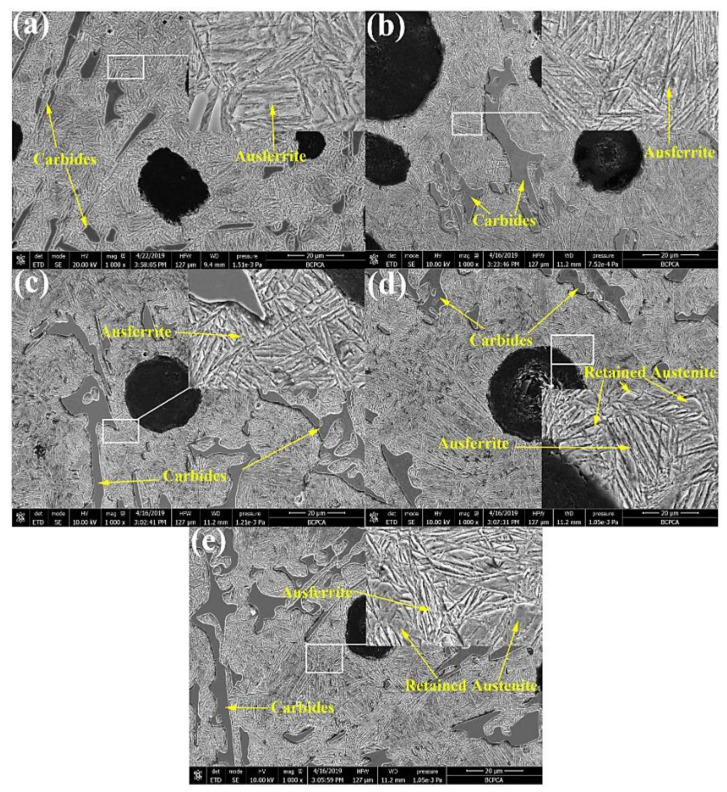
Morphological structures of carbidic austempered ductile iron with varying Cu percentages: (**a**) 0 wt.% Cu, (**b**) 0.5 wt.% Cu, (**c**) 1 wt.% Cu, (**d**) 1.5 wt.% Cu, and (**e**) 2 wt.% Cu [40].

Yang et al. (2020) [40] studied varying Cu percentages mixed with carbidic austempered ductile iron (CADI) in order to increase corrosion resistance. They noticed that the morphology of CADI comprised graphite nodules and carbides, and the matrix comprised bainitic ferrite laths and retained austenite (Figure 12). It was reported that an increase in the Cu content of the composite also increased the retained austenite, and when the Cu content reached 2 wt.%, a bulk-like retained austenite was formed in the matrix (Figure 12e).

**Figure 13:**
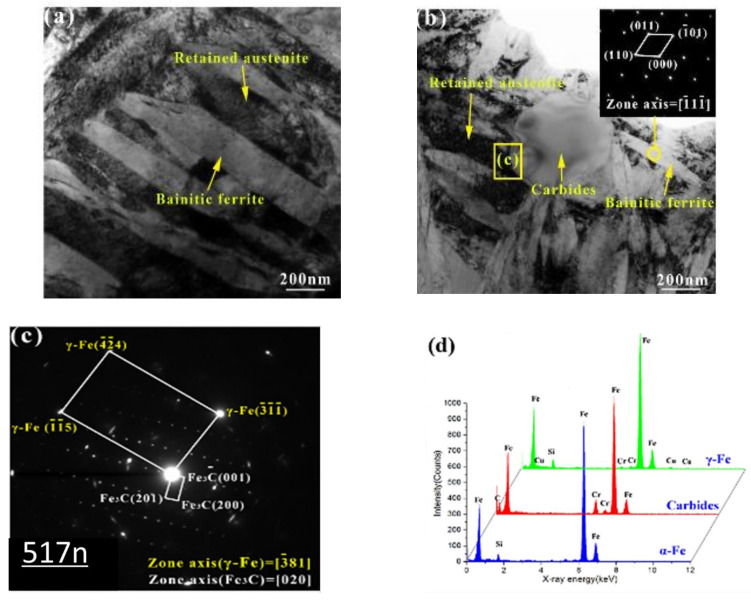
Further morphological structures of Cu-bearing carbidic austempered ductile iron through TEM: (**a**) Cu-free CADI; (**b**) CADI with 2.0 wt.% Cu; (**c**) selected area diffraction patterns (SADPs) of area C in Panel (**b**); (**d**) EDX outcome for three phases [40].

**Figure 14:**
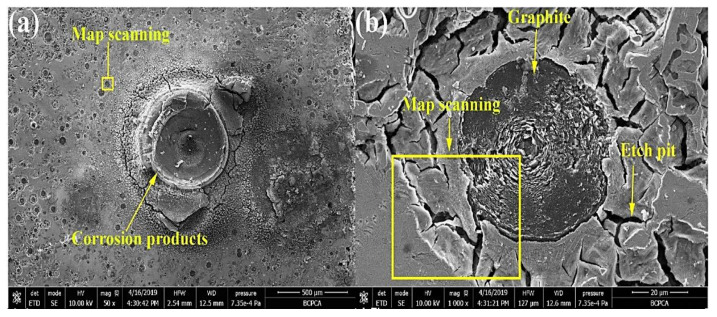
Morphological structures of carbidic austempered ductile iron after corrosion in an acidic environment solution [40]. (**a**) body-centered cubic (BCC) structure, (**b**) face-centered cubic (FCC) structure.

A transmission electron microscope (TEM) was further used to view the formed Cu element, as well as the morphologies of Cu-free and Cu-bearing CADI, as shown in Figure 13. It was observed in the results that the broadness of bainitic ferrite in CADI decreased as 2 wt.% Cu was added. This was due to the fact that a higher amount of Cu content inhibited ferrite formation by preventing C element movement in CADI. It was also observed that bainitic ferrite had a body-centered cubic (BCC) structure, and the retained austenite had a face-centered cubic (FCC) structure (Figure 14). Moreover, it was detected that there was no existence of a Cu-rich compound in Cu-bearing CADI. They concluded that, at 1.0 wt.% of Cu in CADI, corrosive wear resistance both without and with load were optimal under alkaline conditions. However, under acidic conditions, CADI with 1.5 wt.% Cu had the maximum corrosive wear resistance without load.

**Figure 15:**
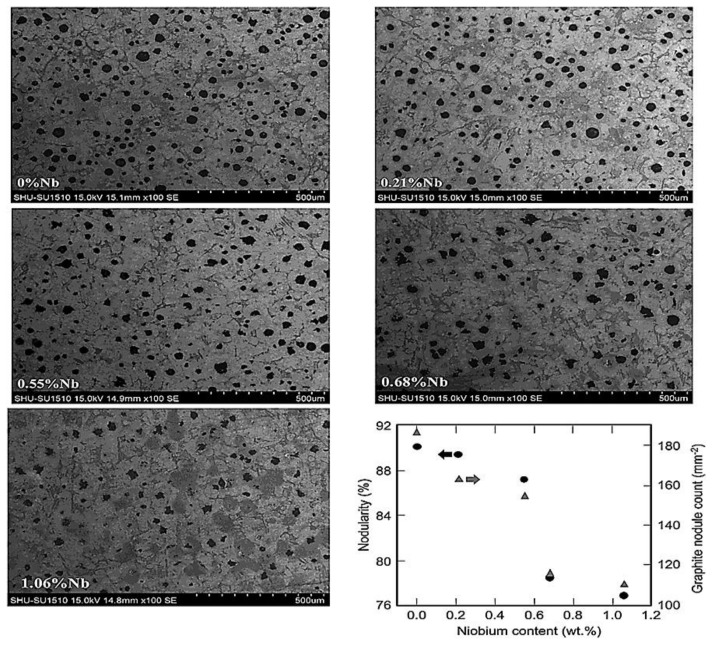
Scanning electron microscope images of graphite in as-cast ductile iron for varying niobium contents and quantitative evaluation of the nodularity and nodule count [41].

The effect of niobium (Nb) alloying on microstructure, the toughness, and the wear resistance of austempered ductile iron was reported by Chen et al. (2019) [41]. They studied the microstructures of graphite and bainite, the bainite transformation process, and properties under austempering conditions. Figure 15, which represents the graphite morphology in the as-cast state, attests that, as the niobium addition increased, the graphite morphology and nodularity decreased. The temperature and kinetics of pearlite and bainite transformation and refinement were affected by Nb inclusion.

**Figure 16:**
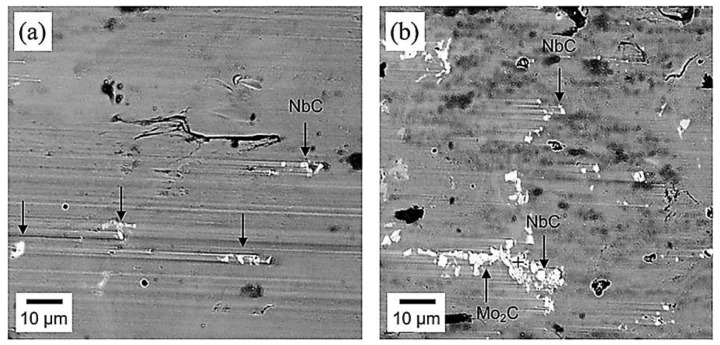
Morphological structures of wear tracks in niobium-alloyed ADI showing (**a**) the impediment of ploughing by distributed niobium-carbon particles and (**b**) big eutectic carbides produced by molybdenum separation in the interfacial region [41].

Figure 16 shows morphological structures of wear patterns in 0.55-percent-niobium-alloyed austempered ductile iron. Niobium carbide was used to make the embedded particles when the scratch surfaces were interrupted (Figure 16a). Because of the significant separation of molybdenum, bigger eutectic carbides composed mainly of molybdenum carbide could be seen in the intercellular region (Figure 16b). The NbC particles were more evenly distributed and existed within the eutectic cells, indicating that they were created prior to eutectic solidification, and they concluded that niobium affected the microstructure of the end-product during all phases of ADI processing, hence changing its mechanical properties.

**Figure 17:**
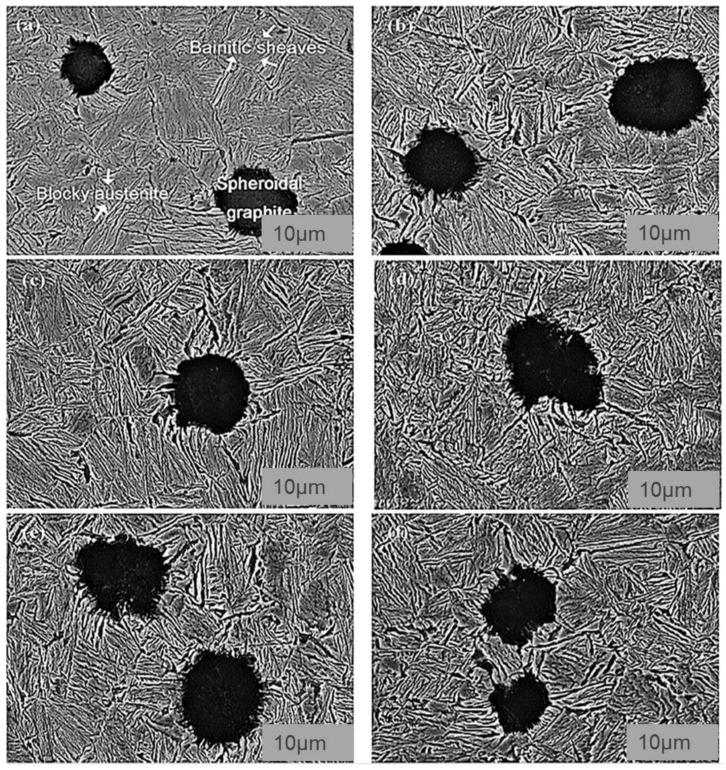
Morphological structures of austempered ductile iron specimens after partitioning at (**a**) 200 °C for 10 min, (**b**) 300 °C for 10 min, (**c**) 200 °C for 30 min, (**d**) 300 °C for 30 min, (**e**) 200 °C for 60 min, and (**f**) 300 °C for 60 min [42].

**Figure 18:**
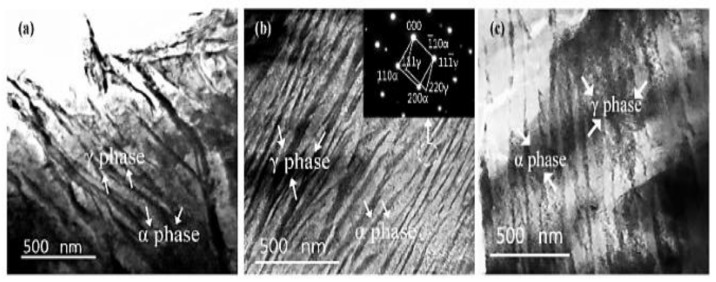
Typical TEM images of (**a**) austempered ductile iron specimen, (**b**) austempered ductile iron specimen after partitioning at 200 °C for 30 min, and (**c**) austempered ductile iron specimen after partitioning at 300 °C for 30 min [42].

Wang et al. (2021) [42] also studied the implication of partitioning heat treatment on the microstructure and mechanical properties of ADI. The morphologies of the austempered ductile iron samples after partitioning heat treatment are shown in Figure 17. Spheroidal graphite, leaf-shaped bainite, and stabilized austenite were detected in all of the samples. A closer examination, on the other hand, revealed differences in bainitic sheave thickness at various temperatures and holding durations. Figure 18 reveals the TEM images of the ADI samples following partitioned treatment. A normal lamellar microstructure could be seen in all of the samples. In the top right corner of Figure 18b, the selected-area electron diffraction (SAED) is shown in the circle areas. Furthermore, no carbides were found through TEM (Figure 18b,c), demonstrating that the partitioning treatment did not produce carbide precipitation. The width of acicular ferrite rose from −40 nm for ADI (Figure 18a) to −80 nm for the partitioned samples (Figure 18b,c), indicating that carbon particles were moved from highly concentrated ferrite to austenite, which resulted in a dispersion in the microstructure after partitioning.

**Figure 19:**
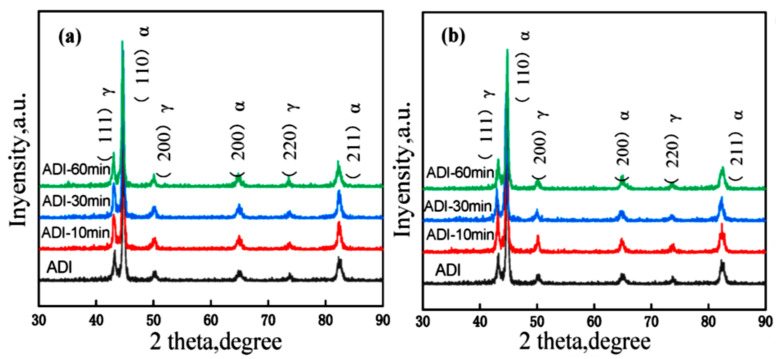
Crystallographic structure of austempered ductile iron specimens partitioned at (**a**) 200 °C and (**b**) 300 °C [42].

The crystallographic structure of the specimens showed that there was no appreciable variation in the intensities of the diffraction peaks for ADI specimens with varying partitioning values, implying that each of the specimens had an equivalent percentage of stable austenite (Figure 19).

**Figure 20:**
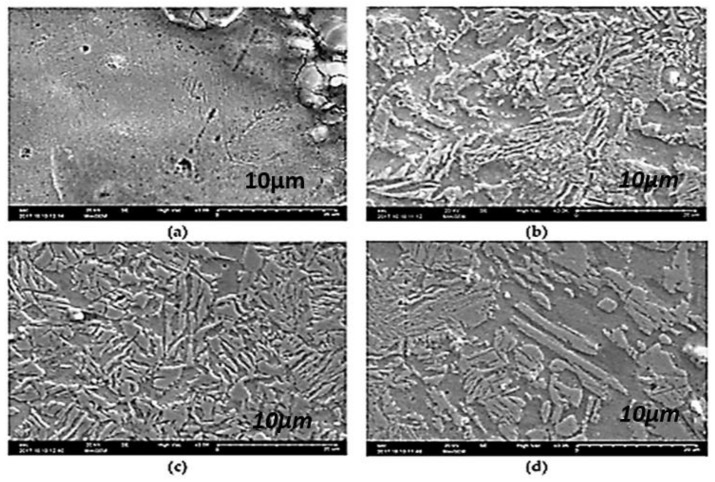
The morphological structures of austempered specimens at varying temperatures: (**a**) 300 °C, (**b**) 320 °C, (**c**) 340 °C, and (**d**) 360 °C [34].

**Figure 21:**
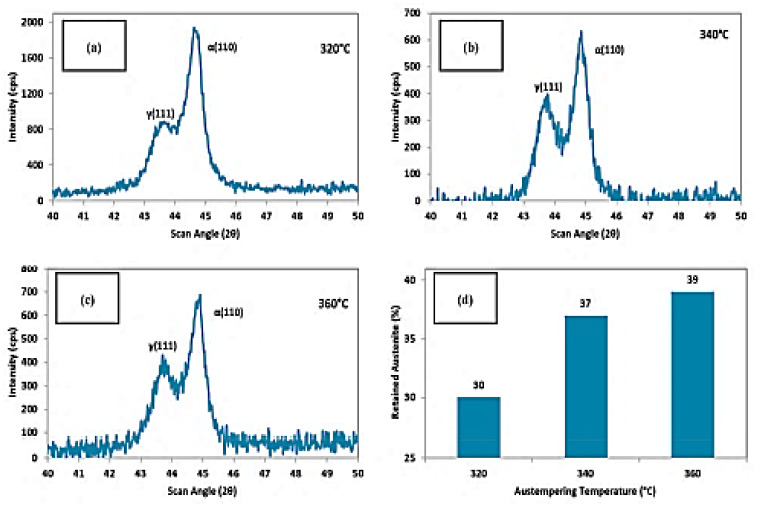
The crystallographic structures at (**a**) 320 °C, (**b**) 340 °C, and (**c**) 360 °C and (**d**) the retained austenite content [34].

The microstructure and mechanical properties of austempered ductile cast iron (ADI) were reported by Sellamuthu et al. (2018) [34] at four trial temperatures of 300 °C, 320 °C, 340 °C, and 360 °C. Figure 20 shows the microstructures of ADI at varying temperatures. Austenite developed at the granular interface of ferrite and, subsequently, expanded to ferrite through nucleation and growth mechanisms. The process of dissolving carbon ahead of ferrite became harder as austenite grew richer and more saturated with carbon. As a result, ferrite growth was slowed. It was also discovered that, as the austempering temperature rose, the microstructure became coarser. The peak locations and intensity planes were determined using crystallographic structure profiles, and the ferrite and austenite contents were computed and are presented in Figure 21. Figure 21d depicts the relationship between austenite content and austempering temperature. As the austempering temperature rose, the amount of retained austenite increased. There was no formation of ausferrite at a lower austempering temperature (320 °C), leaving just retained austenite and lower bainite; however, at higher temperatures, the ausferrite reaction formed at the topmost bainitic area, leaving only reacted austenite in the matrix.

Panneerselvam et al. (2017) [43] studied the effects of the heat treatment process on the microstructure and mechanical features of low-alloyed austempered ductile cast iron with the chemical composition tabulated below. The microstructure of the graphite was surrounded by ferrite, which had a lattice structure with repeating layers of ferrite and cementite. With 85% nodularity, the graphite in pure form seemed circular in shape.

**Figure 22:**
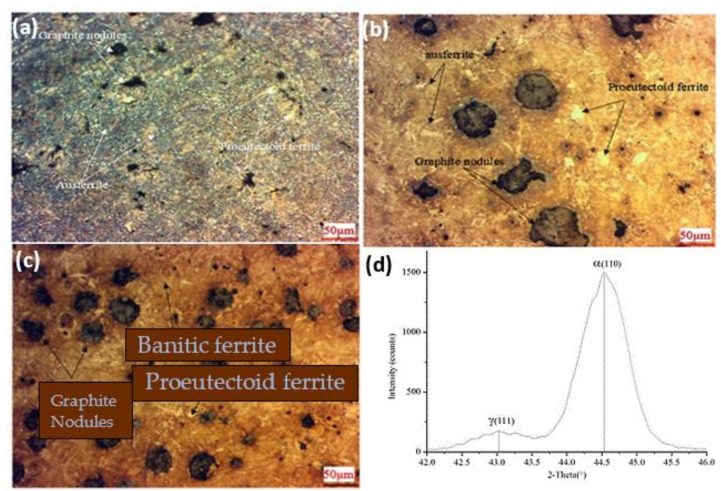
Morphological structure of the ductile iron after heat treatment at 1520 °F and austempering at (**a**) 725 °F, (**b**) 600 °F, and (**c**) 550 °F; (**d**) crystallographic structure of at 550 °F [43].

The visual microstructural images of the austempered specimens at varying temperature are displayed in Figure 22a–c. Table 4 above shows the chemical composition of ADI. The specimens austempered at elevated temperatures had bigger ausferritic microstructures in the matrix, as well as a substantial portion of proeutectoid ferrite. As previously stated, ferritic grain growth is accomplished through a nucleation and growth process. Pearlite reduces as proeutectoid ferrite nucleates, and grain development occurs when heated over a lower critical temperature. Because grain development is influenced by temperature, lower austempering temperatures resulted in more ferrite grains, a smaller volume proportion of austenite in the chemical composition, low carbon austenite yields, and smaller ferritic cell size at standard austempering temperatures. When traditional ADI was austempered at temperatures over the upper threshold, the ferritic cell size was, typically, substantially bigger. It had a wide crystal structure (Figure 22d), where the top austenite was substantially wider and the volume percentage of austenite was lower than the finer ferrite grains. It also had an effect on the interpretation of austenite’s carbon content. They concluded that proeutectoid ferrite could be produced in the matrix microstructure by intercritical austempering. In the matrix, a lower austenitizing temperature resulted in more proeutectoid ferrite.

**Figure 23:**
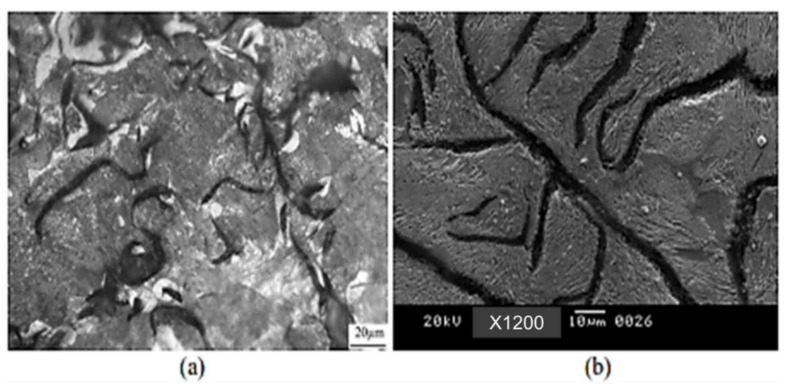
(**a**) Visual microstructure and (**b**) morphological structure of as-cast gray iron [44].

**Figure 24:**
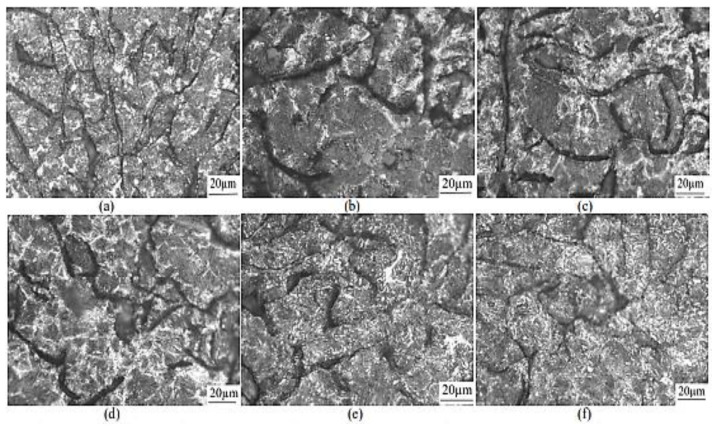
Microstructures of specimens for 1 h at different austempering temperatures: (**a**) 260 °C, (**b**) 285 °C, (**c**) 310 °C, (**d**) 335 °C, (**e**) 360 °C, and (**f**) 385 °C [44].

**Figure 25:**
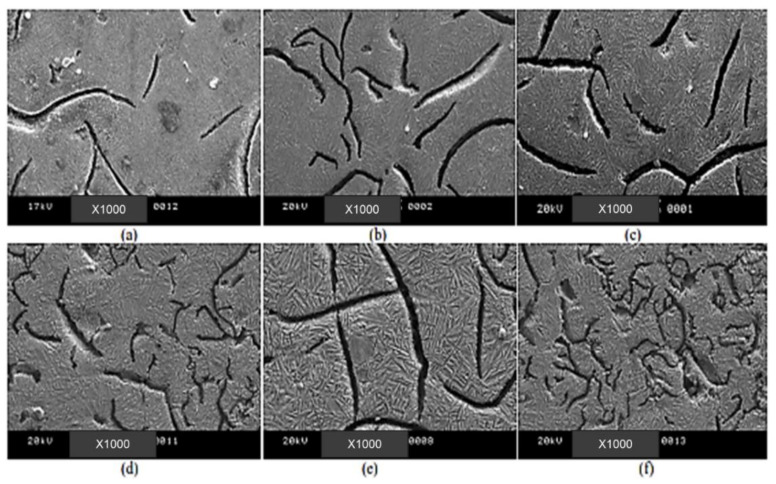
Morphological structures of specimens for 1 h at different austempering temperatures: (**a**) 260 °C, (**b**) 285 °C, (**c**) 310 °C, (**d**) 335 °C, (**e**) 360 °C, and (**f**) 385 °C [44].

**Figure 26:**
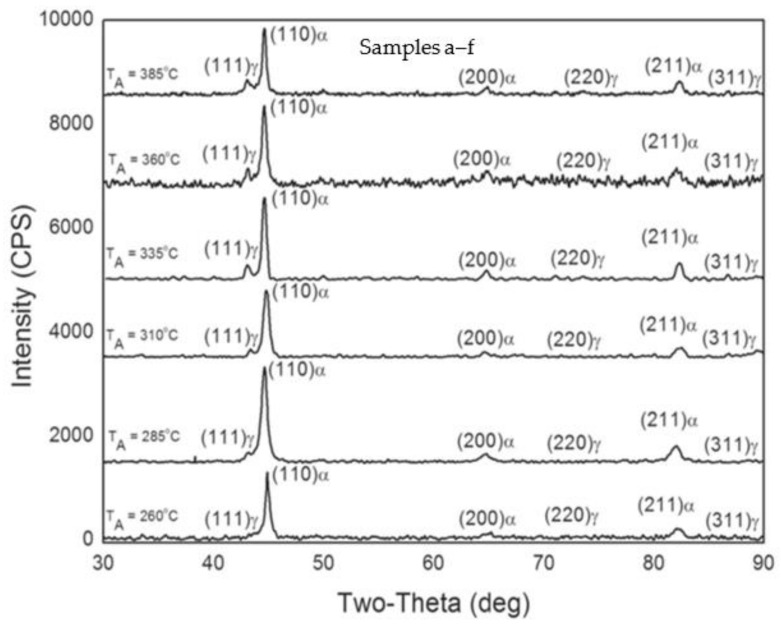
Crystallographic structural analysis of specimens at varying austempering temperatures for an hour: (a) 260 °C, (b) 285 °C, (c) 310 °C, (d) 335 °C, (e) 360 °C, and (f) 385 °C [44].

Sarkar et al. (2018) [44] also studied the mechanical and tribological properties of copper-alloyed austempered gray cast iron at varying austempering temperatures. In the as-cast condition, Figure 23a,b reveals a gray iron microstructure consisting of pearlitic material, ferrite, or a mixture of both. As illustrated in Figure 24a–f and Figure 25a–f, the ADI samples had a mostly lamellar graphite microstructure. The microstructure of the ADI samples was significantly influenced by the austempering temperature. In the microstructure, the dark etching needles were bainitic ferritic, whereas the light etching matrix was a combination of austenite and martensite. Coarse ferritic needles appeared when the austempering temperature rose, indicating that contact with the austenite phase was increasing [45]. The images from the microstructure structure and morphological structure provide a qualitative representation of the microstructural development. The crystallographic structure, as illustrated in Figure 26, provided quantitative information (Figure 26a–f).

**Figure 27:**
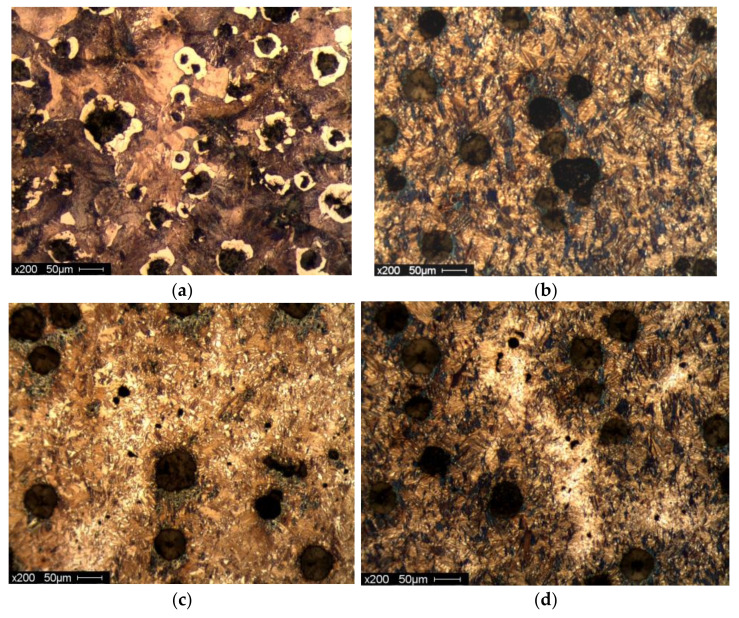
Optical micrographs of (**a**) as-cast sample; (**b**) 10 min at 275 °C; (**c**) 60 min at 350 °C; and (**d**) 60 min at 325 °C.

Nasir et al., 2011 researched heat treatment–microstructure–mechanical and tribological property relationships in austempered ductile iron [46]. In their study, it was observed that the “as-cast” austempered ductile iron sample in Figure 27a contained ductile iron that was primarily pearlitic (85%), ferrite, and nodular graphite. The name “Cow’s Eye” is frequently used to describe the bright ferrite zones that surround the dark graphite nodules. The pearlite-ferrite microstructure in the austempered samples transitioned to ausferrite, martensite, and preserved austenite phases. There were three different types of microstructures. The majority of the material was martensite at lower austempering temperatures (275, 300 °C) (Figure 27b), with a lesser quantity of preserved austenite [46]. It was ausferrite (Figure 27c) at high austempering temperatures (350, 375 °C), with larger portions of retained austenite (27–36%). The martensite needles were dispersed throughout the ausferrite in a mixed ausferrite-martensite microstructure at an intermediate temperature (325 °C) (Figure 27d). Small carbide “islands” (Fe_3_C) were formed inside the “dried-river”-like residual austenite zones. By preventing the development of pearlite during austempering, alloying metals such as Si, Ni, Mo, and Cu helped preserve the carbon-enriched retained austenite. Depending on the austempering time and temperature, austenite created during the austenitizing step changed into martensite, ausferrite, or stable austenite. As a result of the prolonged time for carbon diffusion to turn austenite into high-carbon retained austenite and bainitic ferrite, which was phase one of the transformation, the higher temperature and longer time samples had more austenite. A longer austempering period also enabled the phase two, or “carbide islands,” transition, in which the high-carbon retained austenite further fractured into ferrite and embrittling cementite (Fe_3_C). Avoiding carbide formation during austenitization is one of the key goals of alloying elements [47,48,49].

**Figure 28:**
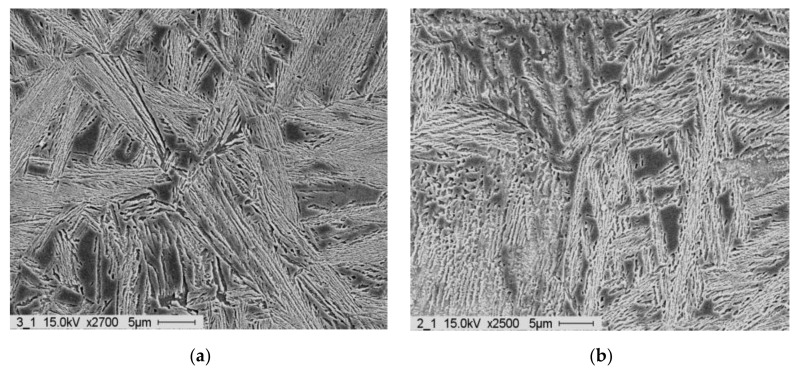
SEM micrographs of (**a**) medium feathery ausferrite and (**b**) highly feathery ausferrite.

The microstructure was also significantly impacted by the austempering period. The martensitic samples had relatively tiny needles in the 60 min samples but coarse needles in the 10 min and 150 min samples. When ausferritic samples were austempered for extended periods of time (60–150 min), the ausferrite tended to be more feathery and less feathery, respectively (10 min). Additionally, the preserved austenite rivers in 150 min samples had more carbide particles. The medium (325 °C, 10 min) and highly (350 °C, 60 min) feathered ausferrite microstructures are compared in Figure 28a,b.

## 5. Effect of Heat Treatment on Tribological Behavior of ADI

**Figure 29:**
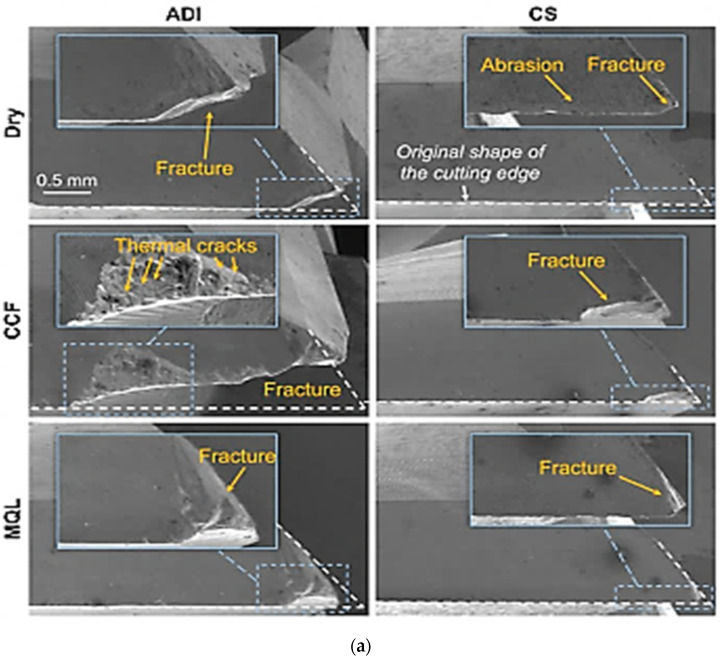

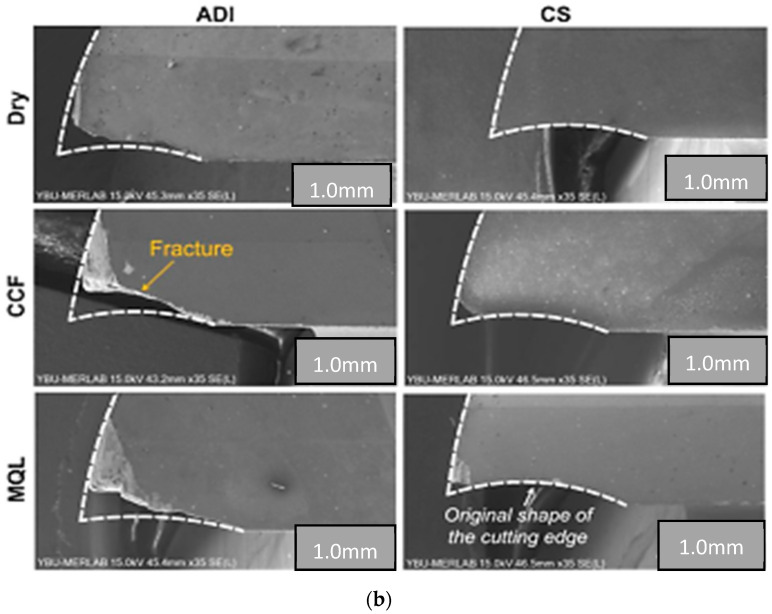
(**a**) Wearing patterns on cutting edges under various milling states [50]. (**b**) Wearing patterns on surfaces under various milling states [50].

Eraslan et al. (2021) [50] studied the effect of heat treatment on the tribological properties of austempered ductile iron and cast steel. The morphological structures of cutting equipment on the edges and surfaces after milling austempered ductile iron and cast steel specimens under various states (dry, conventional cutting fluid, minimum-quantity lubrication) were analyzed (Figure 29). The results revealed that, in the process of milling the specimens, several cracks were generated on both cutting edges and surfaces of the equipment, mostly in conventional cutting fluid and minimum-quantity lubrication states. It was also revealed that the wear mechanism observed was adhesive in nature, and the wear rate in milling cast steel under a dry state was lower than the austempered ductile iron.

The influence of austempering temperature on the wear rate of austempered ductile iron was studied by Du et al. (2020) [39], and they discovered that the wearing rate of the specimen raised as there was increment in the load. At high stress, the ADIs wore at similar rates; however, under low load, the wearing rate raised as the austempering temperature increased. At low load, the wearing rate of the ADI at 380 °C was slightly higher than that under high load. Furthermore, as the load was raised, the wearing rate of the identical specimen rose. The effectiveness of the friction coefficient in reducing the wear rate was also seen by checking the difference between the wearing rates of ADIs austempered at 380 °C under low and high loads.

**Figure 30:**
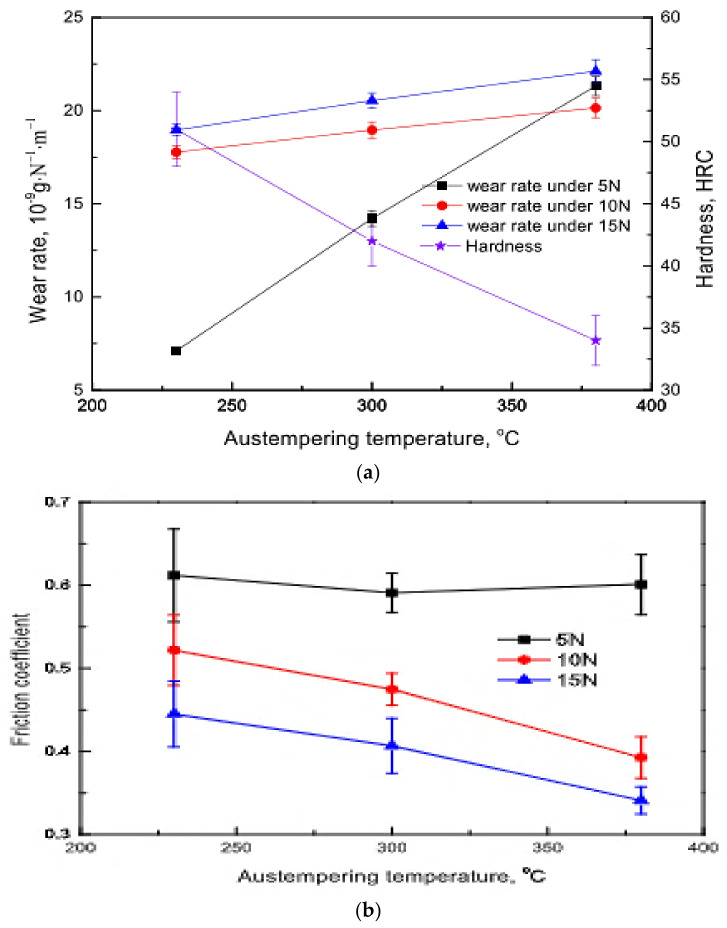
(**a**) The wearing and hardness of ADI ranges with austempering temperature under varying forces [39]. (**b**) The ranges of coefficient of friction of samples vs austempering temperature [39].

It can be observed in Figure 30b that increase in the friction coefficient at the initial state translated to a steady state after small fluctuations. One characteristic that was noticeable was that the friction coefficient reduced as there was increment in the load ball used on the disk for the tribometer machine. The friction coefficient also decreased with increasing austempering temperature at high load but increased slightly under low load.

**Figure 31:**
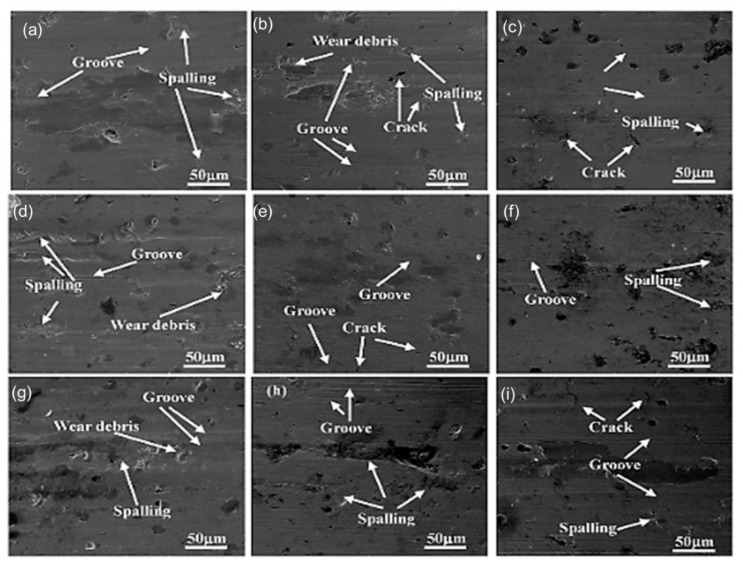
The morphological structures of worn samples austempered at varying temperatures and forces: (**a**) 230 °C, 5 N; (**b**) 230 °C, 10 N; (**c**) 230 °C, 15 N; (**d**) 300 °C, 5 N; (**e**) 300 °C, 10 N; (**f**) 300 °C, 15 N; (**g**) 380 °C, 5 N; (**h**) 380 °C, 10 N; (**i**) 380 °C, 15 N [39].

The morphology of the worn surface after it dried was examined (Figure 31). Grooves and spalling covered the worn surface, showing that abrasive and adhesive wear were the primary wearing processes for ADI. It also revealed fissures within the cracks and wear debris dispersed across the worn surface.

**Figure 32:**
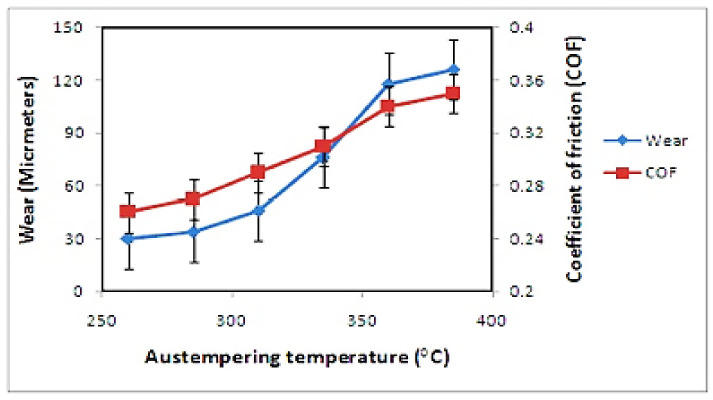
Effectiveness of austempering temperature on tribological behavior of the samples [44].

The tribological behavior of ADI as a result of varying austempering temperature (Figure 32) was studied by Sarkar et al. 2018 [44]. It was reported that wearing rate decreased with decrease in the friction coefficient at a low austempering temperature. However, at a greater austempering temperature, the wearing rate decreased with increase in the friction coefficient. The wear values ranged from 30 to 126 micrometers, while the coefficient of friction (COF) values ranged from 0.26 to 0.36. It was discovered that, as the austempering temperature rose, wearing rate increased and the fiction coefficient rose.

**Figure 33:**
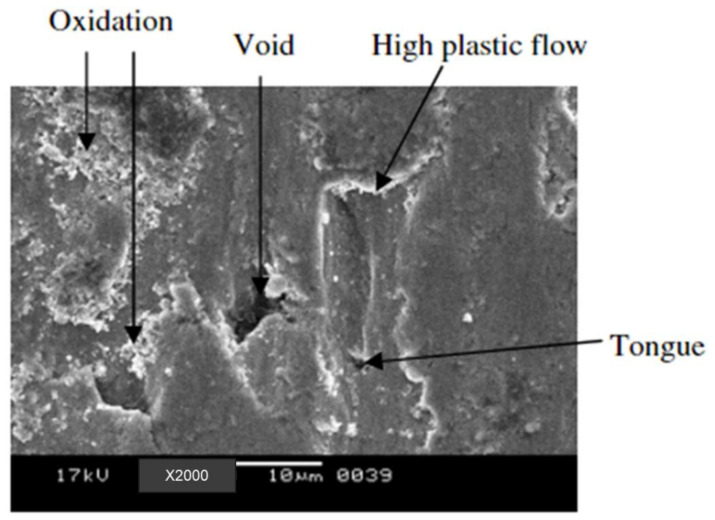
Worn surface micrograph of as-cast sample [44].

**Figure 34:**
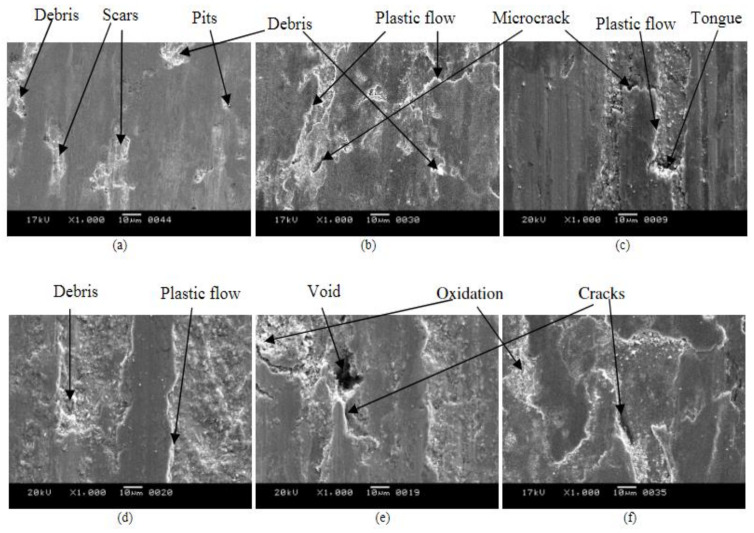
Worn surface micrographs of ADI samples at different austempered temperatures for 1 h: (**a**) 260 °C, (**b**) 285 °C, (**c**) 310 °C, (**d**) 335 °C, (**e**) 360 °C, and (**f**) 385 °C [44].

The morphologies of the worn surfaces of the as-cast (Figure 33) and austempered samples (Figure 34) were studied. The wear surfaces of the ADI samples consisted mostly of deep wear marks with fewer pitting and elastic flows at lower austempering temperatures. The surface look changed dramatically as the austempering temperature rose, revealing traces of scratching and microcracks. There was proof of considerable elastic flows as the temperature rose (e.g., 360 °C, 385 °C), as illustrated in Figure 34e,f.

**Figure 35:**
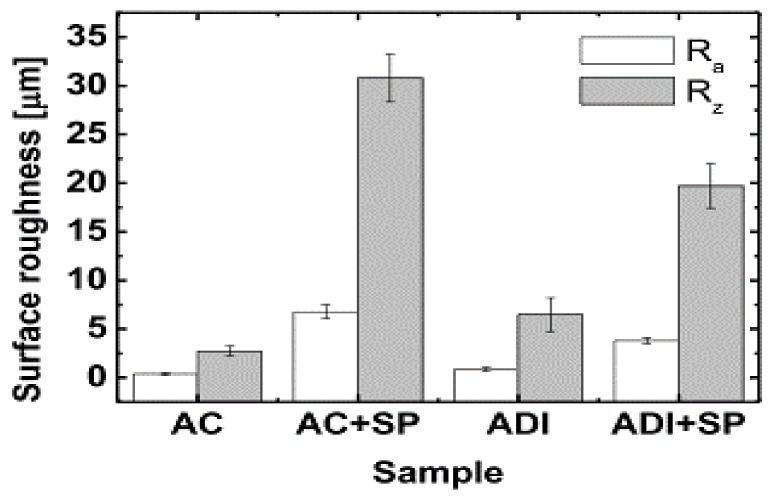
Microhardness variables obtained during shot peening on cast and ADI samples [51].

**Figure 36:**
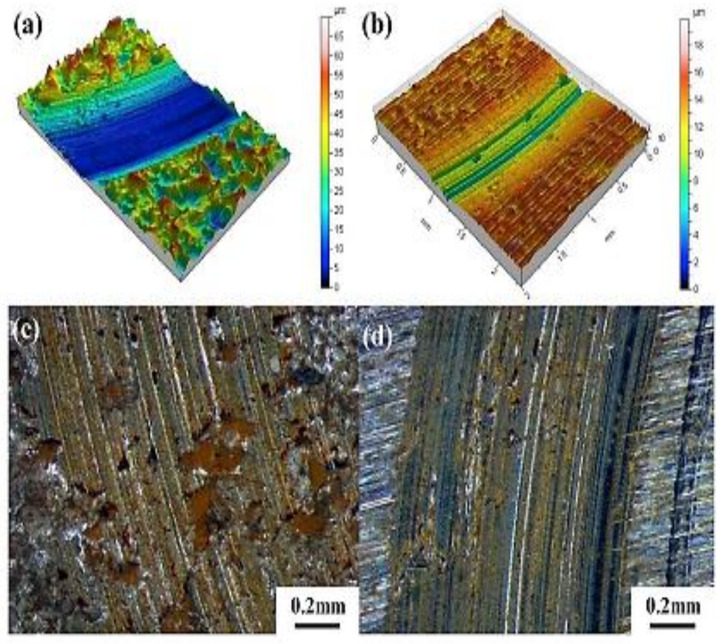
The microstructures of the worn patterns acquired during ball-on-disk testing on the as-cast samples (**a**,**c**) and austempered samples (**b**,**d**) [51].

The effect of shot peening on the residual tribological behavior of cast and austempered ductile iron was reported by Silva et al. (2019) [51]. The results of the surface roughness (R) measurements obtained from their experiment are shown in Figure 35. It was observed that the shot-peened samples showed the maximum roughness. The cast material had an average roughness of 0.37 μm, which increased to 6.77 μm after peening of shots (AC + SP), whereas the austempered samples had an initial roughness of 0.87 μm and later increased to 4.23 μm after peening of shots. The worn surface micrographs of the specimens were examined, and it was concluded that the shot-peened samples had higher wear resistance than the unpeened samples. (Figure 36a,b).

Different researchers [52,53,54,55] have previously demonstrated that modifying the austempering process parameters (time and temperature) can change the microstructure of ADI and lead to various wear behaviors. In the current investigation, it was determined that this was accurate. As previously indicated, the theory of microst ructural evolution can be used to explain this phenomenon. Finer ferritic cell size (d) and greater volume fractions of austenite (γ) and austenitic carbon (X_γ_C_γ_) in the matrix are created using a two-step austempering process. Higher hardness and yield strength, but lower ductility and tensile toughness, are the improved mechanical qualities of ADI as a result of these microstructural parameters. Therefore, the microstructure and mechanical characteristics have an impact on ADI tribological behavior, particularly its abrasion wear behavior.

**Figure 37:**
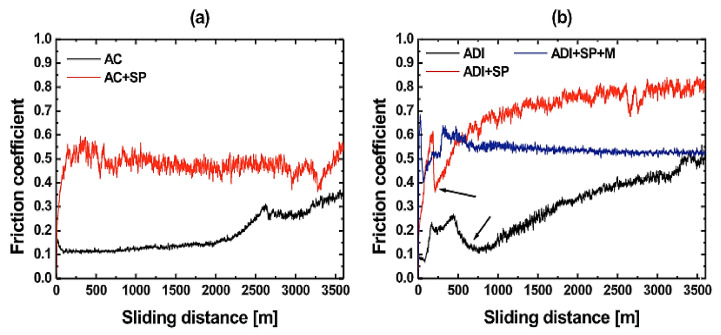
Shows the variation in wear resistance as a result of wear loss for (**a**) as-cast and (**b**) austempered samples [51].

**Figure 38:**
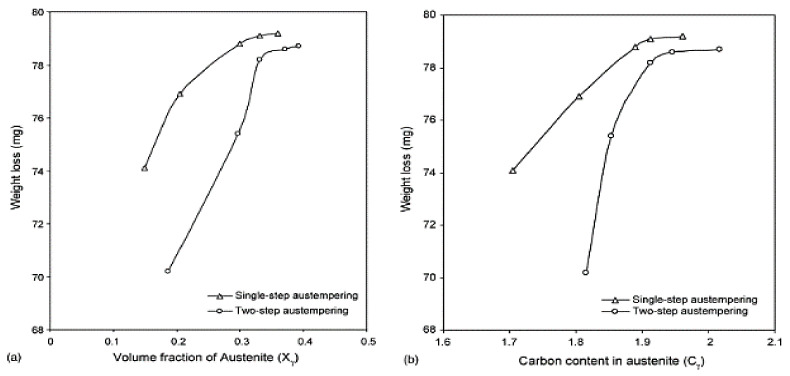

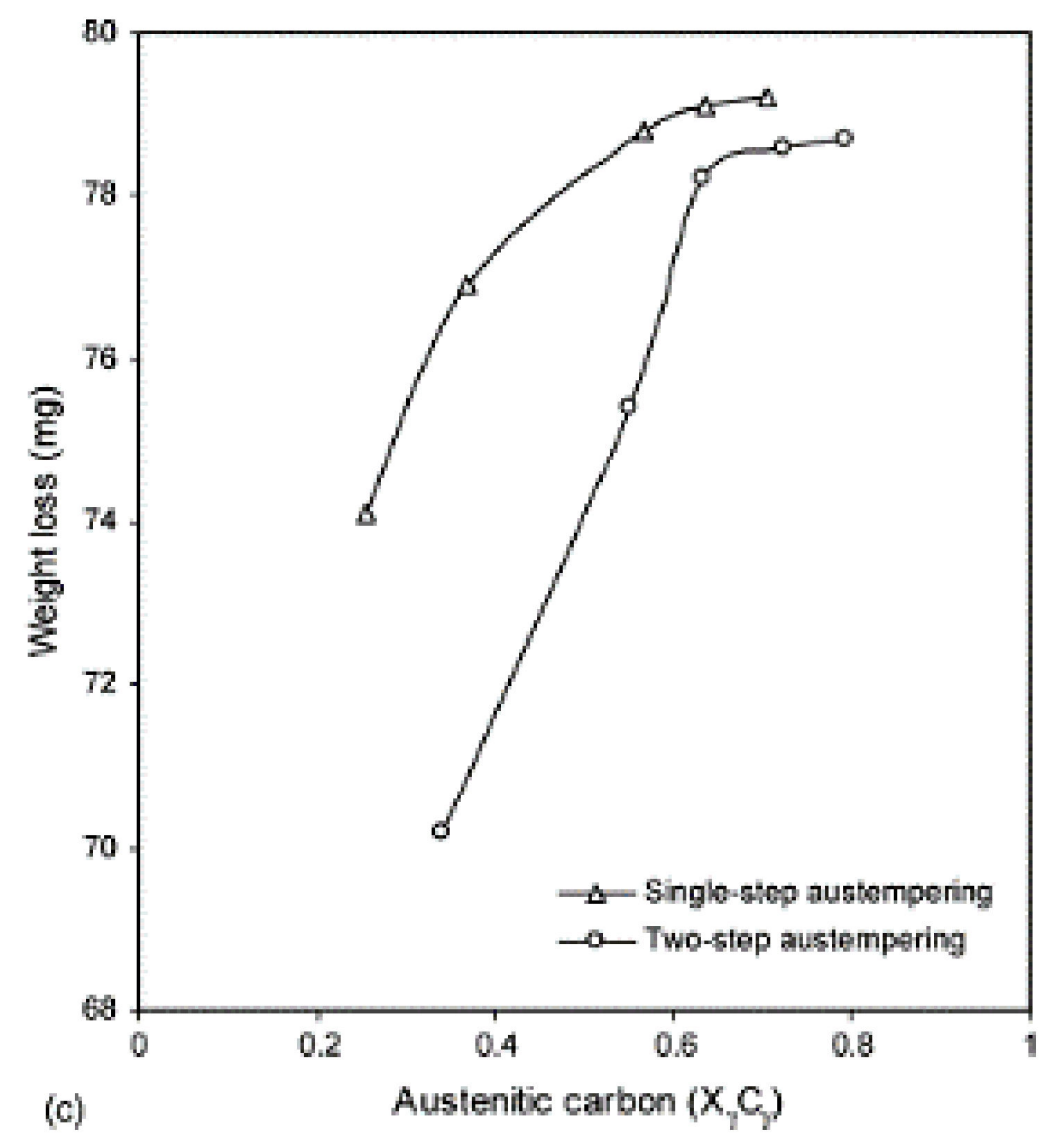
A graph of weight loss from wear against ADI microstructural characteristics: (**a**) austenitic volume fraction (X_y_), (**b**) austenite carbon content (C_y_), and (**c**) austenitic carbon (X_y_C_y_).

The impact of microstructural factors on ADI wear behavior is depicted in Figure 38a–c. The weight loss of the ADI after the wear test is plotted against (a) austenite volume fraction (X_y_); (b) austenite carbon content (C_y_); and (c) austenitic carbon (X_y_C_y_) in Figure 37a,b. It is clear from Figure 38a that the wear loss increased as the volume percentage of austenite (X) increased. However, two-step-treated samples had less weight loss for the same volume percentage of austenite. With a more ductile and softer phase than BCC ferrite, austenite featured an FCC lattice structure and a faster rate of strain hardening. Accordingly, the wear resistance should decrease as austenite’s proportion in the microstructure increased.

## 6. Effect of Heat Treatment on Mechanical Behavior of ADI

**Figure 39:**
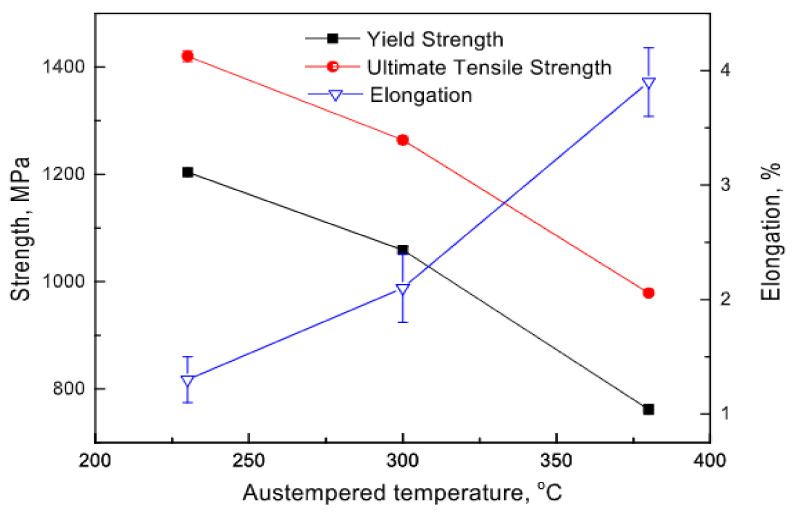
The mechanical variable at different austempered temperatures [39].

The effectiveness of austempering temperature on the mechanical behavior of austempered ductile iron was studied by Du et al. (2020) [39]. It was observed that the ductility of the austempered ductile iron was discovered to rise when the austempering temperature decreased, and the ultimate tensile strength of the specimens was observed at a lower austempering temperature for a specific period of time (Figure 39).

**Figure 40:**
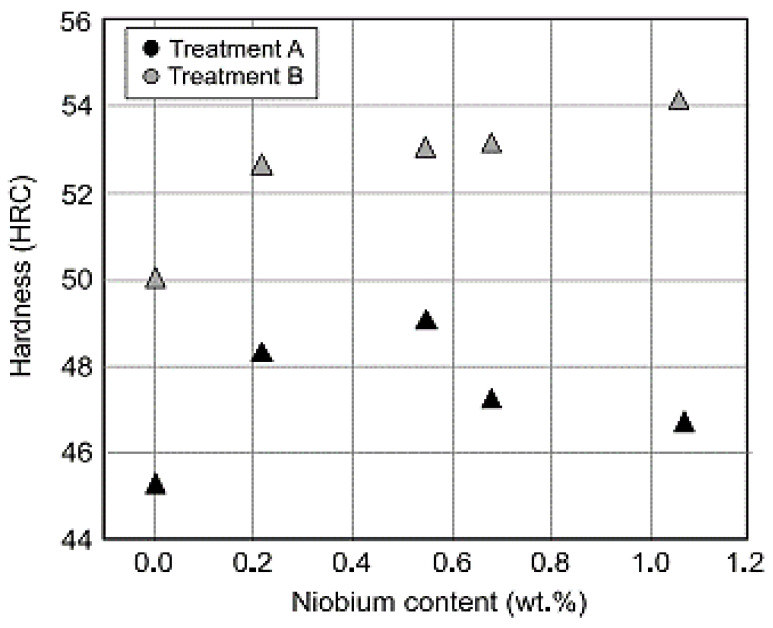
The effectiveness of niobium inclusion and austempering temperature on hardness of the samples [41].

**Figure 41:**
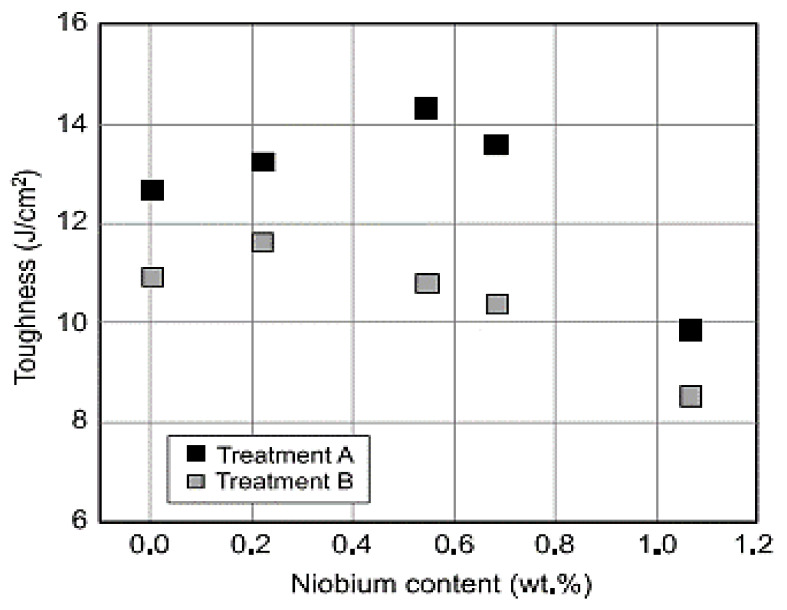
The effectiveness of niobium inclusion and austempering temperature on shock resistance of the samples [41].

The mechanical properties of the niobium alloy in austempered ductile iron were reported [41], and it was observed that, as the niobium content increased, so did the hardness and toughness (Figure 40 and Figure 41).

**Figure 42:**
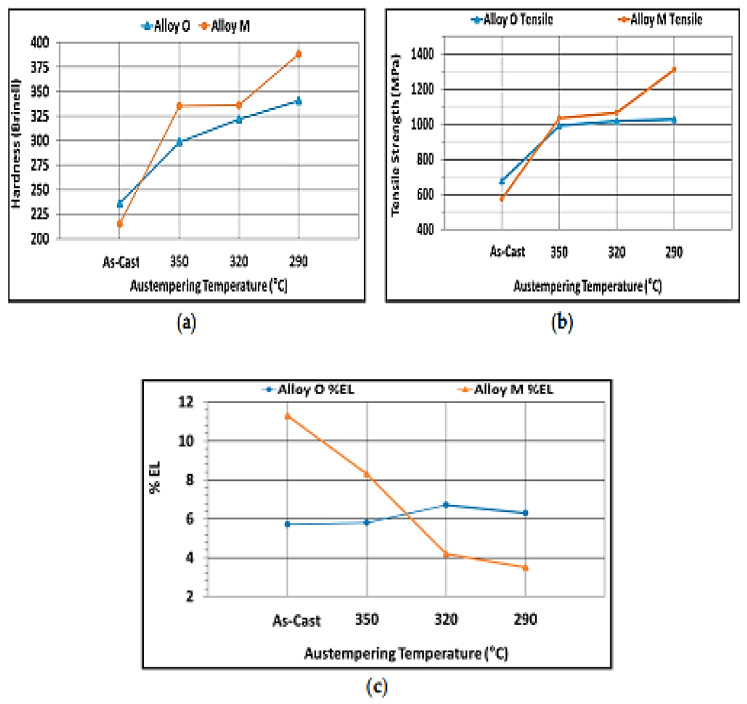
The outcomes of the mechanical properties of the as-cast and austempered specimens [56]. (**a**) Hardness (**b**) Tensile strength, (**c**) Percentage elongation.

It was also reported by Konca et al. (2017) [56] that the hardness of the Ni-Cu-alloyed and the Mo-Ni-Cu-alloyed specimens rose by 44 and 80 percent, respectively, as the austempering temperature also rose, while the ductility of the specimens reduced (Figure 42). In addition, the strength of Mo-Ni-Cu-alloyed specimen rose as the ductility and austempering temperature reduced, while the elongation decreased with decrease in the austempering temperature.

**Figure 43:**
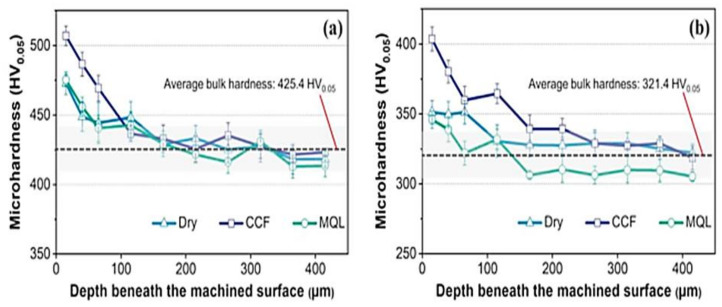
The microhardness below the workpiece surface for (**a**) austempered ductile iron and (**b**) cast steel samples [50].

Eraslan et al. (2021) [50] also observed the increase in subsurface hardness under conventional cutting fluid for austempered ductile iron and cast steel compared to dry and minimum-quantity lubrication states (Figure 43).

## 7. Summary and Recommendation

In order to overcome the brittleness challenges and catastrophic failures encountered by applied loads in present-day applications, after providing some detailed information on the processing techniques, morphological performances, phase analyses, and mechanical properties of cast iron families with relevance to potential mechanisms, the following conclusion are made, as well as recommendations.

### 7.1. Morphological Properties

Austempering temperature and a shorter holding period could be used to manufacture needle-like ferrite platelets for austempered ductile iron (ADI) and other graphite cast irons. Upon raising the temperature of austempering conditions or prolonging the holding period, ferrite grows more coarse. Nonetheless, if indeed the second step of the austempering process is started, it is likely that the carbon-saturated austenite may break down into bainitic ferrite and carbide.

### 7.2. Mechanical Properties

With the development of ausferrite that resembles feathers, the hardness of graphite cast irons, such as ADI, DI, and GCI, and their respective tensile strengths were decreased in terms of mechanical performance. The introduction of unique austempering conditions could improve the ferritic platelets in all the developed cast irons, boost the volume of carbon-saturated austenite, and raise the carbon content of stable austenite. Compared to other cast iron products, austempered ductile irons had superior mechanical characteristics. When the austempering temperature or holding time was raised, the hardness and tensile strength of all the products of austempered ductile irons deteriorated. This was due to the presence of some carbide precipitations.

### 7.3. Tribological View

Due to the decrease in hardness observed, the tribological behaviors of cast iron products, such as DI, GCI, and ADI, degraded at high austempering temperatures and prolonged holding times. The production process could lead to decreased wear loss under equivalent hardness compared to conventional materials, such as carbon steel and cast irons generated by isothermal conditions. The superior surface hardness and stress-induced transformation of residual austenite into martensite were linked to exceptional wear resistance. Additionally, conventional surface hardening techniques, such as shot peening, laser hardening, and the inclusion of nano-size particles, could improve the wear resistance of single-step ADI even more. Abrasive wear, oxidative wear, adhesive wear, delamination, and fatigue wear were all included in a comprehensive wear mechanism. As anticipated, the wear behavior of austempered ductile irons had superior properties compared to ductile and gray cast irons. This was ascribed to the enhanced stress-induced transformation reaction during the production process, which was enhanced by the mechanical characteristics and refined ferrite platelets. On the one hand, bench testing or actual working situations, as opposed to laboratory tests alone, should be used to examine the tribological properties of cast iron products. Additionally, more emphasis should be placed on the development of mathematical models.

## Figures and Tables

**Table 1:** Ranges of chemical composition for DI casting [21].

Elements	Ferritic Grades (%)	Pearlitic Grades (%)	Functions	Remarks
C	3.00–4.00	3.00–4.00	Spheroidal constituent	Accumulation leads to graphite formation
Si	1.80–3.00	1.80–2.75	It functions as both graphitizer and ferritizer	It also leads to serious regulation of controls for as-cast pearlite
Mg	0.03–0.06	0.03–0.06	It serve as a spheroidizer	Excess encourages carbides
Ce	0.030 max	0.30 max	When combined with Mg, it promotes the formation of spheroids	Excess encourages carbides
S	0.015 max	0.015 max	Combines with Mg	Sulfur removal is critical
Mn	0.20 max	0.70 max *	Pearlite former	Excess encourages carbides
P	0.035 max	0.05 max	Embrittles structure	Stabilizer for pearlite
Cr	0.040 max	0.10 max	Very effective carbide former	Annealing is hindered
Cu	0.03 max	To specification	Strength and hardness	Pearlite stabilizer with high potency

NB *: Because of the effect on carbide formation, it is not recommended to exceed 0.3% Mn in pearlite grade.

**Table 2:** Different grades of austempered ductile iron and the corresponding mechanical properties.

Identification Grade	American Standard Grade Based on ASTM89	Ultimate Tensile Strength (Mpa)	Strength at Yield (Mpa)	Percentage of Elongation (%)	Range of Hardness (in Brinell Standard)
A	130-90-09	895.70	620.10	9	269–341
B	150-110-07	1033.50	757.90	7	302–375
C	175-125-04	1205.75	861.25	4	341–444
D	200-155-02	1378.00	1067.95	2	388–477
E	230-185-1	1584.70	1274.65	1	402–522

**Table 3:** Different types of austempered ductile iron and their mechanical properties as designated by ASTM A897-A897M-06.

Identification Grade	American Standard Grade Based on ASTM89	Ultimate Tensile Strength (MPa)	Strength at Yield (MPa)	Percentage of Elongation (%)	Impact Energy (J)	Range of Hardness (in Brinell Standard)
A	750-500-11	750	500	11	110	241–302
B	900-650-9	900	650	9	100	269–341
C	1050-750-7	1050	750	7	80	302–375
D	1200-850-4	1200	850	4	60	341–444
E	1400-1100-2	1400	1100	2	35	388–477
F	1600-1300-1	1600	1300	1	20	402–512

**Table 4:** Chemical composition of austempered ductile cast iron.

Elements	C	Si	Ni	Cu	Mn	Cr	Mg	Al	V	P	Ce	Ti	Mo	S	Sb	Sn
Composition (% by mass)	3.44	2.46	1.03	0.52	0.08	0.05	0.043	0.018	0.017	0.016	0.013	0.01	<0.01	0.008	<0.005	<0.005

## Data Availability

The study did not report any data.
